# Sense of belonging within the graduate community of a research-focused STEM department: Quantitative assessment using a visual narrative and item response theory

**DOI:** 10.1371/journal.pone.0233431

**Published:** 2020-05-21

**Authors:** Christiane N. Stachl, Anne M. Baranger

**Affiliations:** 1 Department of Chemistry, University of California, Berkeley, California, United States of America; 2 Graduate Group in Science and Mathematics Education, University of California, Berkeley, California, United States of America; University of Lleida, SPAIN

## Abstract

It is well-documented that the representation of women and racial/ethnic minorities diminishes at higher levels of academia, particularly in science, technology, engineering, and math (STEM). Sense of belonging—the extent to which an individual believes they are accepted, valued, and included in a community—is often emphasized as an important predictor of retention throughout academia. While literature addressing undergraduate sense of belonging is abundant, there has been little investigation of sense of belonging in graduate communities. Because graduate training is required to generate new scientific leaders, it is important to understand and address sense of belonging at the graduate level—paying explicit attention to devising strategies that can be used to increase representation at higher levels of academia. Here, a visual narrative survey and item response modeling are used to quantify sense of belonging among graduate students, postdoctoral researchers, and faculty within the Department of Chemistry at the University of California, Berkeley. Results suggest that graduate students, postdoctoral researchers, and faculty all experience impostor phenomenon. Respondents struggle most with maintaining positive self-perceptions of their productivity, capabilities as a scientist, and success—particularly in comparison to their peers. Communicating about science with peers, talking about teaching hurdles, and engaging in mentoring relationships also contribute significantly to sense of belonging. Faculty members have the highest sense of belonging, while senior graduate students and postdoctoral researchers are least likely to feel a sense of belonging. Additionally, graduate students and postdoctoral researchers who identify as underrepresented, as well as those who submit manuscripts for publication less than their peers, are less likely to feel a sense of belonging. This is the first study to generate a quantitative, nuanced understanding of sense of belonging within the entire graduate academic community of an R1 STEM department. We envision that these methods can be implemented within any research-focused academic unit to better understand the challenges facing community members and identify factors to address in promoting positive culture change. Furthermore, these methods and results should provide a foundation for devising interventions that academic stakeholders can use to directly improve graduate education.

## Introduction

The underrepresentation of women and some racial and ethnic groups remains a significant, global issue in science, technology, engineering, and math (STEM) fields [1–3]. Although progress is being made to increase diversity in STEM, it is well-documented that the representation of these groups diminishes at higher levels of academia [2,4,5–12,13]. One factor that is often emphasized as an important predictor of success and retention in academia is feeling a sense of belonging within one’s field of study [14–18]. Sense of belonging is defined as the extent to which a person believes that they are accepted and included as a legitimate member of an academic community and that their presence and contributions to that community are valued [16,19–21]. It encompasses the sociopsychological aspects of academic membership that are not directly related to intellect—such as a sense of shared identity, social connectedness with peers, and mental health [22]—and is known to negatively impact persistence among women and underrepresented minorities (URMs) in STEM [6,16,19,23–30].

### Theoretical framework

Sense of belonging in the academic context is not a novel or unique phenomenon. The need-to-belong theory suggests that humans maintain an inherent desire and motivation to form and maintain interpersonal relationships, regardless of the context [31]. Similar to hunger or personal safety, this basic human need to belong also influences behavior [32]—a lack of belonging or social exclusion can lead to anxiety, stress, and depression [33,34]. Thus, the implications of low sense of belonging have motivated much social and behavioral research on academic membership.

In undergraduate STEM populations, for example, low sense of belonging is correlated with low academic achievement and self-efficacy [26,35], feelings of ‘being an outsider’ and needing to ‘fragment’ one’s identity in order to fit in [36,37], and even impostor phenomenon—the belief that success results from luck, working harder than others, or manipulating other people’s impressions, rather than through genuine ability [38,39]. One study conducted on first-year undergraduate students found that sense of belonging can be explained by five categories: perceived peer and faculty support (how comfortable a student feels reaching out to peers and faculty for help, and whether they feel those individuals are open to helping them); class comfort (how comfortable a student is asserting opinions sharing ideas in the classroom); perceived isolation (social connectedness on a personal level); and empathetic faculty understanding (should difficult situations arise) [40]. Literature also suggests that the extant, negative stereotypes regarding women’s ability and expectations for success [19,23,37], in addition to the persisting lack of female role models and mentors [41], affects sense of belonging among undergraduate women and negatively impacts their persistence in STEM [5,16,30,19,23–29].

Individuals transitioning to higher levels of academia face new challenges. The feelings of isolation and uncertainty that can emerge due to the rigor of graduate academic culture affect belonging [11,37]. Yet, research focused on understanding sense of belonging among graduate students and faculty members is uncommon [32,42]. The small body of existing literature that describes graduate student sense of belonging suggests that because graduate students spend most of their time within their department and laboratory (rather than in residence halls and across campus, as do undergraduates), their sense of belonging is more connected to the few faculty mentors they have, whom they depend on to provide guidance on their professional development and scientific identity [43–45]. A study completed to understand whether the five factors that affect sense of belonging in first-year undergraduates also influence sense of belonging in graduate students found that graduate student sense of belonging is more affected by investment in education, the need to balance education with life demands, friendships with peers, and relationships with faculty [46]. One other study found that while professional relationships, microaggressions, and microaffirmations influence graduate student sense of belonging, the general lack of professional networks and role models carries more weight in shaping graduate student sense of belonging [42].

### Motivation

Doctoral training is required to generate new scientific leaders. Importantly, an individual’s graduate education experience can heavily influence their decision to remain in academia, pursue a high-rank career in industry or elsewhere, or leave STEM altogether. Still, research aimed at understanding sense of belonging disproportionately focuses on undergraduate student populations. Moreover, the few existing studies concerning graduate student sense of belonging are limited in scope. They either focus solely on understanding singular aspects of academic membership—such as publication rate [47], program structure [30], or mentoring and professional networks [42]—or rely on small interviewee populations to gather more information about factors that affect sense of belonging [45,46]. In addition, only a small number of studies have suggested practical ways to combat the factors that contribute to low sense of belonging in graduate communities. For example, Fisher et al. suggest making graduate program expectations and performance standards more rigid and clear to students, to reduce academic disparities [30]. However, given that the academic environments of individual departments are unique, it is critical to develop a method to assess and address aspects of graduate academic climate at the department-level that impact sense of belonging. Once a substantive understanding of sense of belonging is generated, it can be used to devise interventions for directly addressing the factors that negatively affect academic sense of belonging [1,48–50].

### Design choice

This is the first research study to use a visual narrative survey and Item Response Theory (IRT) to quantify sense of belonging among graduate students, postdoctoral researchers, and faculty. The visual narrative format of the sense of belonging survey makes it possible to convey naturally-occurring facial expressions, bodily postures, and social interactions in every survey item, thus supporting the comprehension of meaning, place, and context that text alone cannot always elicit [51–54]. In addition, IRT analysis has the following distinct advantages: it produces a detailed, quantitative ordering of the aspects of graduate education and academic culture (survey items) that contribute the most or least to graduate student sense of belonging (central construct); IRT is suitable for use in large-scale assessment of sense of belonging; it enables the analysis to be conducted with any subset of respondents based on a target variable, such as year or division in the program, to provide detailed information regarding the aspects of belonging that influence any subset of respondents the most; it calibrates item ‘difficulties’ and respondent abilities on the same, linear scale [55], which enables analysis of longitudinal changes in respondent sense of belonging that may be due to institutional efforts to improve academic culture over time; and overall, IRT enables identification of the unique aspects of academic culture that contribute the most and the least to sense of belonging—a benefit which allows this methodology to be generalized for any academic department or institution.

### Research questions

Our research questions for this study are as follows:

1. Can we identify aspects of the academic culture and climate within an R1 Chemistry Department that correlate positively and negatively with student sense of belonging?
2. What are the prominent factors contributing to sense of belonging among graduate students, postdoctoral researchers, and faculty? Are these factors the same or different across respondent populations?
3. How do the factors affecting graduate student sense of belonging compare to those that influence undergraduate student sense of belonging?
4. Does sense of belonging vary by year in graduate school? Or by other academic and demographic factors?

This work, conducted within the University of California, Berkeley Department of Chemistry, allows us to unveil aspects of academic climate and culture that can be addressed to improve sense of belonging within the graduate community of an R1 STEM department. Berkeley Chemistry’s large population of graduate students facilitates large-scale data collection from an ethnically and geographically diverse population that allows general conclusions to be made, which expand the scope of foundational knowledge on sense of belonging. Moreover, because many graduates of the Berkeley Chemistry Ph.D. program ultimately become faculty at top-tier institutions, the results of this work—as well as any interventions developed to address sense of belonging—may propagate throughout academia, informing current and future generations of faculty about the issues that affect sense of belonging within a research-focused graduate community. Overall, this work provides a blueprint by which to assess sense of belonging among the members of any doctorate-granting STEM department and contains important implications for developing strategies to ensure that all department members feel valued, welcome, supported, and included in their academic workplace.

## Methods

### Survey design

The sense of belonging (SB) survey was developed to include dimensions of graduate education that were emphasized in responses to the first Berkeley Department of Chemistry academic climate survey as important for the department to improve on as a community [49], and which are also known to influence undergraduate student sense of belonging [16,21,24–26]. In addition, facets of sense of belonging that surfaced as recurring themes in Department of Chemistry roundtable conversations involving ~20 graduate students, ~2–3 postdoctoral researchers, and ~7 faculty were included in the SB survey. Several rounds of item paneling with ~25 graduate students in the Graduate School of Education were also carried out in the process of developing the SB survey questions.

A pilot study was conducted in which 32 graduate students from either the Graduate School of Education or the Department of Chemistry completed the SB survey. All of these students participated in a follow-up think aloud interview. Interviews with six faculty regarding the pilot survey were also conducted. Results and feedback from the pilot study were used to optimize the effectiveness, usefulness, and clarity of each survey illustration. We relied on extensive item paneling and pilot study interviews to ensure variation in the appearances of the characters (skin color, hair type and color, clothing, and corresponding positively- or negatively-worded statement), to minimize implicit bias within the visual narrative.

**Fig 1:**
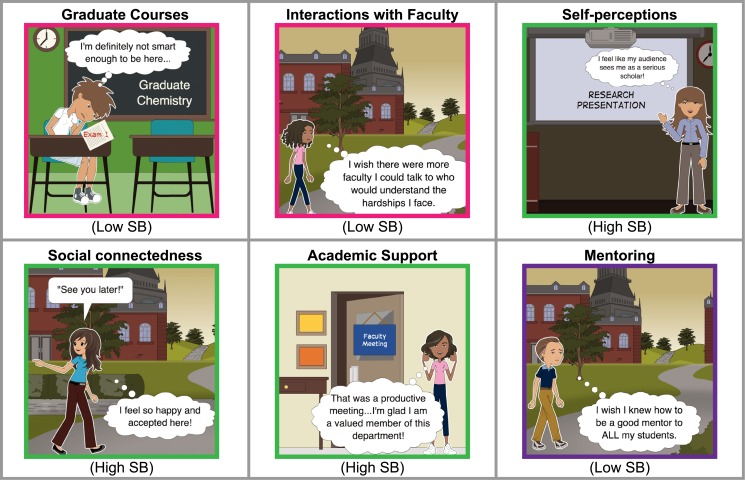
Examples of the sense of belonging (SB) survey illustrations. Illustrations that are unique to the graduate student and postdoctoral researcher SB survey are outlined in pink, illustrations that are unique to the faculty SB survey are outlined in purple, and illustrations that are worded to convey the same context in both SB surveys are outlined in green. The characters in the faculty SB survey are portrayed as visually older than those in the graduate student and postdoctoral researcher SB survey.

Some of the dimensions of sense of belonging, which were brought up by faculty, graduate students, and postdoctoral researchers in the process of developing this survey, have not been addressed in existing studies of sense of belonging among graduate students [30,42,45–47]. The final SB surveys include a total of 15 illustrations for graduate students and postdoctoral researchers, and 12 for faculty members. The illustrations fall under the following categories: graduate coursework, teaching, mentoring, interactions with peers and faculty, academic support from peers and mentors, social connectedness, self-perceptions with respect to ‘undefined’ measures of success (self-perceived intelligence, productivity, competence, independence, and value), and the frequency of submitting manuscripts for publication relative to lab mates and same-career stage peers. The latter illustrations exist only and were placed last in the graduate student and postdoctoral researcher survey, in order to avoid them affecting respondents’ attitude toward or responses to other illustrations. The faculty SB survey illustrations depict scenarios that parallel those in the graduate student and postdoctoral researcher SB survey, but which are suited to issues and context that are appropriate for faculty. Six illustrations are worded such that the context is identical across both SB surveys. The faculty and graduate student/postdoctoral researcher SB surveys included a balance of negative- and positive-worded illustrations, in order to account for differences in how respondents may interpret them [56]. Examples of the SB survey illustrations are provided in Fig 1. Each illustration is color-coded by whether the illustration belonged to the graduate student and postdoctoral researcher (pink) or faculty (purple) SB survey, or both (green). The full SB surveys are provided in **Fig A in** S1 Text; all illustrations were designed using Pixton Comics Inc.© (pixton.com).

The prompt associated with each illustration is the same: “*Please indicate to what extent each of following cartoons relates with your current experience in the Department of Chemistry*.” All questions were forced-choice, 5-point Likert-scale items: ‘*do not relate’; ‘rarely relate’; ‘sometimes relate’; ‘often relate’; and ‘always relate’*; and included a *‘prefer not to respond’* answer choice. Respondents were also given the option to explain their response *via* an open-ended text box.

### Sense of belonging construct

In order to quantitatively measure sense of belonging using IRT, every survey item (question) should relate back to a central construct (sense of belonging) [55]. Additionally, all possible response choices for every survey item should map onto this central construct, creating a continuous, 5-tiered “scale” of sense of belonging (Table 1).

**Table 1:** Sense of belonging (SB) construct and scoring guide.

Positively-Worded (“High SB”^a^)	Negatively-Worded (“Low SB”^a^)	Overall SB Score	SB Construct
Likert-Scale Items	Likert-Scale Items		
Always Relate [to cartoon]	Do Not Relate [to cartoon]	**4**	**Highest Belonging**
			*Respondent feels all or a combination of the following*: *smart*, *productive*, *successful*, *valued*, *accepted*, *that they belong*, *that they have faculty they identify with*, *that they have a supportive social network*, *that they are a competent scientist*
Often Relate [to cartoon]	Rarely Relate [to cartoon]	**3**	**High Belonging**
Sometimes Relate [to cartoon]	Sometimes Relate [to cartoon]	**2**	**Neutral**
			*Respondent (1) relates with feeling a sense of belonging ~50% of the time*, *or (2) experiences some factors that contribute to sense of belonging but not others*
Rarely Relate [to cartoon]	Often Relate [to cartoon]	**1**	**Low Belonging**
Do Not Relate [to cartoon]	Always Relate [to cartoon]	**0**	**No Belonging**
			*Respondent feels all or a combination of the following*: *unintelligent*, *unproductive*, *unsuccessful*, *undervalued*, *that they do not belong*, *that they do not have faculty they identify with*, *that they do not have a supportive social network they enjoy being around*, *that they are not a competent scientist*

^a^(“High SB”) and (“Low SB”) descriptors correspond to the illustrations in the SB survey that are positively- and negatively-worded, and which map directly and inversely onto the overall sense of belonging construct, respectively.

Half of the illustrations in each SB survey are positively-worded (“High SB”) and half are negatively-worded (“Low SB”)—these descriptors are present below each illustration in Fig 1 and **Fig A in** S1 Text. Positively- and negatively-worded items map directly and inversely onto the overall sense of belonging construct, respectively (left-hand columns in Table 1), and the latter are reverse-coded accordingly. To aide with interpretation of data from positively- and negatively-worded items, the negatively-worded narratives are re-worded throughout this paper to map onto the SB construct. The original and corresponding, re-worded narratives are listed in **Table A in** S1 Text.

### Survey administration

The SB surveys were administered confidentially to graduate student, postdoctoral, and faculty researchers in the Department of Chemistry using Qualtrics LLC ©, as an addendum to the 2019 annual academic climate survey [49]. Upon completion of the 2019 academic climate survey [49], respondents were prompted to either (1) proceed to the additional, separate, SB survey or (2) exit the Qualtrics platform. Completion of both surveys was voluntary—respondents were instructed to skip any question(s) they did not feel comfortable answering. The Qualtrics "anonymize responses" function was used to retroactively delete all potentially identifying information from survey responses. To incentivize participation, flyers indicating survey dates and details were posted around the UC Berkeley College of Chemistry. Additionally, two $100 gift cards a business of choice were offered to any participant who filled out the survey(s) and elected to enter their name voluntarily in a prize drawing. To maintain confidentiality, this drawing was conducted in a separate, online Google Form such that respondent emails could not be associated with their survey responses. This study was authorized by the University of California, Berkeley institutional review board, approval/Protocol ID: #2019-01-11732. All participants completed informed consent.

### Population

The total response rate from Department of Chemistry graduate community members was 34.1%. This includes 49% of graduate students, 22% of postdoctoral researchers, and 21% of faculty. 42% of student or postdoc survey respondents identified as female, indicating that men and women filled out the survey at roughly equal rates, as approximately 40% of the department is female. **Table B in** S1 Text contains the total numbers of respondents and members of the department. The following demographic information was collected: gender, self-identity as belonging to an underrepresented group (URG), international student status, department division (synthetic chemistry, physical chemistry, or chemical biology), and year in Ph.D. program. No demographic information other than department division was collected for faculty members, as the low overall numbers of faculty in the Department of Chemistry might compromise the confidentiality of responses.

Of graduate student and postdoctoral researcher respondents, 56.1% identified as belonging to Underrepresented Groups (URGs). While this number is high, our definition of URG is broad—it includes, but is not limited to, individuals that identify as female; being from underrepresented racial, religious, ethnic, sexual orientation, and international groups; having disabilities (defined as those with a physical or mental impairment that substantially limits one or more major life activities); and having a low socio-economic status [57]. Given the underrepresentation of women and racial/ethnic minority scholars in STEM, the term URG was used to enable a general comparison of URG and majority respondent populations, while still maintaining a balanced representation of study participants. Racial/ethnic minority respondent responses are not analyzed separately due to concerns about compromised confidentiality from low total numbers of these students and faculty in the department.

### Data scoring

Data scoring incorporated open-ended text responses and Likert-scale responses; Likert-scale data was scored based on the sense of belonging scoring guide and construct in Table 1, and the *please explain* open-ended text responses were used to gain insight into the justification of each respondent’s level of relation to each illustration. Blank and *prefer not to respond* answers, as well as comments that contained no relevant information with respect to the illustration are considered “missing data”. All respondent comments referred to throughout this text were obtained from open-ended responses.

### Item response theory analysis

The item response model used here is the partial credit model, which is suitable for analysis of responses to ordered polytomous items (questions), such as Likert-scale items, that are scored at two or more levels [58–61]. The partial credit model is unidimensional (i.e., measures a single latent trait, like sense of belonging), probabilistic (i.e., models the probabilistic relationship between responses to a survey and the respondent’s ‘ability’—how much of the latent trait they have), and assumes monotonicity of the construct [62]. For the purpose of our analysis, this suggests that as a respondents’ sense of belonging increases, their probability of positively endorsing an item also increases. Thus, an item that is less likely to be positively endorsed is considered more ‘difficult’, and vice versa.

Mathematically, the partial credit model measures the latent trait (sense of belonging) as a function of respondent ability (their ‘amount’ of belonging) and item difficulty (i.e., an item’s probability of being endorsed) [63,64]. The unit of measurement for respondent ability and item difficulty is the ‘log of odds unit’, generally known as the ‘logit’ [65]. IRT analysis calculates a logit score for every survey respondent and item. The logit score of a respondent represents their ‘ability’ to positively endorse survey items and is related to their raw, overall score on the entire survey. Respondents with higher logit scores are considered to have higher ‘ability’, or sense of belonging. The logit value of an item represents the probability of that item being endorsed. Thus, the more positive the logit value of an item, the more difficult that item is considered to be—such that items with large, positive logit values require respondents to have ‘more’ sense of belonging in order to endorse those items, and vice versa [66,67].

In practice, the logit scale typically ranges from -3 to +3. A logit value of zero can indicate a respondent or item with ‘average’ ability or difficulty, respectively. Positive logit values represent respondents with greater than average ability, or items that are more difficult to endorse. Negative logit values represent respondents with less than average ability, or items that are ‘easier’, or more likely to be endorsed by the population [66–68]. The probabilistic nature of this measurement model effectively ‘calibrates’ respondent ability and item difficulty onto the same, continuous, logit scale. This allows direct comparison between respondent abilities and item difficulties [68], along a logit scale that maps directly onto the sense of belonging construct in the right-most column of Table 1 [59,69,70].

To fully describe the difficulty of a polytomous item, the partial credit model also calculates the difficulty of each response choice for every question (in Logit units). Each ‘score difficulty’ is indicated by a Thurstonian threshold, which is defined as the ability a respondent needs in order to have a 50% probability of endorsing a given response choice or a more positive one [65]. Items with 5 response choices are described by 4 Thurstonian thresholds, each of which represents a 50% probability of endorsing a score of 1 (rarely relate) or more; 2 (sometimes relate) or more; 3 (often relate) or more; and 4 (always relate), respectively.

The psychometric properties of each SB survey were evaluated using the item response modeling approach [55]. Specific evidence about the reliability and validity of the SB survey instruments is presented in S1 Text, along with the overall difficulty of each item and the corresponding weighted infit mean square (MNSQ) and 95% confidence interval (**Tables D and E in** S1 Text). These data support the conclusion that the visual narrative produces valid and reliable data as a measure of sense of belonging, and provide evidence that each item’s data fit the partial credit model appropriately.

All item response, latent regression, and differential item function analyses were completed using ACER ConQuest: Generalised Item Response Modelling Software, Version 2.0 [58]. All other statistical analyses were completed in Microsoft^®^ Excel for Mac, Version 16.16.9. ConQuest commands for carrying out partial credit model analysis are included in the S1 Text.

## Results

### Wright map: Visualizing sense of belonging among graduate students and postdoctoral researchers

**Fig 2:**
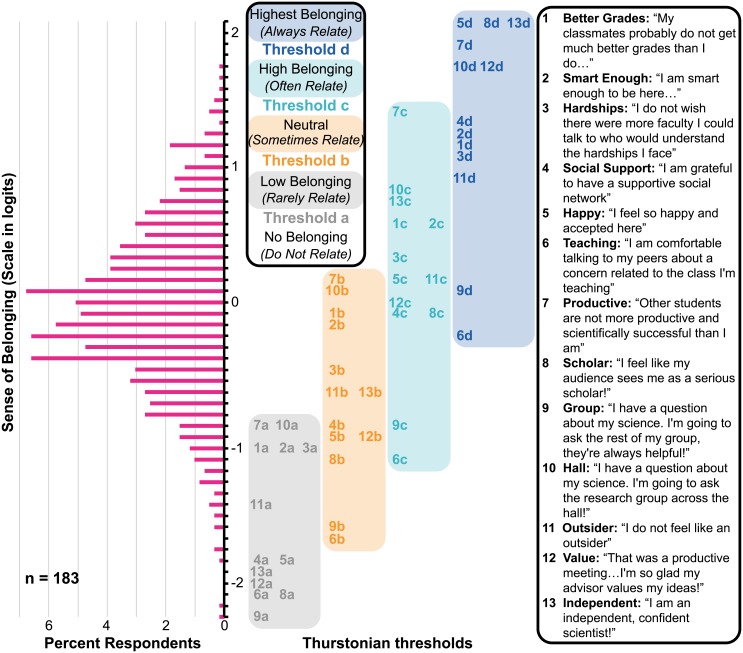
Graduate student and postdoctoral researcher Wright map. The histogram on the left-hand side represents the distribution of respondent scores (in logit units) along the sense of belonging construct (y-axis); respondents that lie toward the top of the histogram have higher sense of belonging (and vice-versa). The Thurstonian thresholds for each SB survey item are shown on the right-hand side and are color-coded and grouped by letter (group ‘a’ represents Thurstonian threshold ‘a’, etc.). The color-coding of the legend represents the four possible ‘score categories’ defined by Thurstonian thresholds. Items are labeled by their item number; the full, positively-worded narrative for each SB survey item is listed on the far right.

Partial credit model analysis generates a “Wright map”—a vertical plot that illustrates the distribution of logit values for respondents and items. The Wright map for the graduate student and postdoctoral respondent data is shown in Fig 2; the y-axis is a logit-unit scale. The left-hand side of the y-axis in Fig 2 contains a histogram of respondent abilities, and the right-hand side of the y-axis presents the item Thurstonian thresholds. There are four Thurstonian thresholds for every survey question: threshold ‘a’ (grey) represents a 50% probability of endorsing a score of 1 (*rarely relate / low sense of belonging*, based on the SB construct in Table 1) or more on any given item; threshold ‘b’ (orange) represents a 50% probability of scoring 2 (*sometimes relate / neutral*) or more; threshold ‘c’ (light blue) represents a 50% probability of scoring 3 (*often relate / high sense of belonging*) or more, and threshold ‘d’ (dark blue) represents a 50% probability of endorsing a score of 4 (*always relate / highest sense of belonging*). The Thurstonian thresholds are grouped and color-coded by their corresponding letter, shown in the legend in the middle of Fig 2.

In order to analyze the Wright map in Fig 2 in its entirety, it is important to note that the y-axis logit scale enables direct comparison of respondent abilities and Thurstonian thresholds, and also relates directly to the sense of belonging construct in the right-most column of Table 1 [55]—respondents that score higher along the y-axis have a higher sense of belonging (ability) as well as a greater probability of scoring at more difficult Thurstonian thresholds. In this way, respondent logit scores can be interpreted with respect to Thurstonian threshold logit values. If a respondent’s logit value is greater than the logit value of a given Thurstonian threshold, then the respondent likely scored in the upper score category represented by that threshold. Thus, respondents who scored toward the top of the histogram in Fig 2 endorsed more (or more difficult) items, and are probable to have higher sense of belonging than those who scored toward the bottom of the histogram. In this way, the Wright map enables direct interpretation of respondent sense of belonging at the item level—the position of a respondent on the histogram indicates the Thurstonian threshold(s) they are probable to have endorsed. In Fig 2 for example, ~1% of respondents have a logit score of -1, and Thurstonian thresholds 1a, 2a, and 3a also have a logit score of -1. This shows that this ~1% percent of respondents scored above thresholds 8b, 6c, 11a, 9b, 6b, 4a, 5a, 13a, 12a, 6a, 8a, and 9a, and have a 50% probability of having endorsed thresholds 1a, 2a, and 3a. No other method of analysis enables such a detailed, direct comparison between respondent scores and item difficulties, making item response analysis a more informative means of understanding sense of belonging within a population.

### The barrier for talking to fellow lab mates and graduate student instructors is low

The majority of respondents always relate with (achieve highest belonging with respect to) items 6 (comfortable talking about teaching concerns) and 9 (comfortable asking groupmates about science), as ≥ 50% of the respondent histogram lies above the Thurstonian threshold d logit value for both items. This propensity for respondents to relate with discussing teaching concerns and talking to their lab mates about science may be due to a positive culture of peer connectedness within research groups and among students while they are teaching. In fact, in open-ended responses, respondents suggested that their lab mates are helpful, collaborative, supportive, and “patient and non-judgmental about [their] questions”.

The Berkeley Chemistry Department requires that all incoming graduate students teach as a Graduate Student Instructor and take a pedagogy course in tandem during their first semester of the program. Respondent comments suggest that having this teaching-network right from the start of the Ph.D. program enables students to feel comfortable speaking about their teaching concerns: “we all taught together our first semester, so that lowered the barrier [to] talking to peers about teaching…it’s a common ground that everyone can relate with or at least empathize with” and “the graduate student community here is pretty supportive in terms of teaching and addressing problems that come up in class.” Additionally, because teaching does not ‘count’ toward graduate student success, students may also feel more comfortable discussing their teaching in a negative tone due to the lower stakes nature of this aspect of graduate education. Traditionally, items like 6 and 9 that maintain consistently low threshold values are considered too ‘easy’ for the respondent population and can be discarded from analysis. However, these data are important to highlight, as they suggest that the Berkeley Chemistry doctoral program successfully emphasizes teaching and destigmatizes difficult conversations regarding teaching experiences.

### Graduate students and postdoctoral researchers find it difficult to ask other research groups about science

Thurstonian threshold values for item 10 (comfortable asking group across the hall about science) are -0.85 (a), 0.14 (b), 0.87 (c), and 1.79 (d) logits. These threshold values are all consistently greater than those of items 6 and 9 (discussed in previous section). In fact, ~75% of the respondent histogram lies below the threshold c logit value for item 10, and the logit value of threshold d is more positive than all respondent logit values. This suggests that respondents did not strongly relate with item 10, such that it is consistently less probable for respondents to endorse feeling any belonging with respect to asking research groups across the hall a question about science. It is interesting to note the contrast between items 9 and 10—at least 50% of respondents strongly relate with asking one’s own research group a question about science, while only ~25% of respondents relate with feeling positively about asking research groups across the hall a question about science. Many respondents commented that they either “feel VERY awkward about [asking a group across the hall]” or do not feel that “another research group was more equipped to answer [my research question]”. Respondents also noted that there is not a lot of collaboration or communication between research groups, suggesting that collaboration within groups is greater than among groups.

### Impostor phenomenon is a prominent aspect of graduate student and postdoctoral researcher sense of belonging

Thurstonian threshold values for item 7 (feel as productive and successful as peers; a: -0.83; b: 0.21; c: 1.41; d: 1.91 logits) are as consistently high as those of item 10 (discussed in previous section). Approximately 90% of the respondent histogram lies below the logit value for threshold c of item 7, indicating that only ~10% of respondents relate with feeling positively about being as productive and scientifically successful as their peers. Rather, most respondents feel *neutral* or negative with respect to their self-perceptions of their productivity and success. In addition, at least 50% of respondents score below threshold c for items 8 (viewed as a serious scholar), 12 (advisor values my ideas), and 13 (independent confident scientist), and the logit value of threshold d for each of these items is more positive than any respondent logit values. This indicates that, in general, respondents do not strongly relate with feeling independent, confident, valued by their advisor(s), or like a serious scholar. In fact, respondents remark in the open-ended comments section of each survey question that they experience impostor syndrome: we are “judged severely based on productivity”; and “we tend to talk more about our successes…[so] the general feeling is that others are getting more done than we are.” Interestingly, many respondents commented that publishing contributes significantly to their stress. Respondents mentioned that not having published by their fourth year of the program, or not having published as much or in the same high impact journals as their peers, leads to them feeling “crappy” and makes it “hard to feel like [they are] working hard enough.” One respondent wrote that “it’s really hard to ignore the impostor syndrome when present[ing] research…I doubt my skills and often think that people watching my presentations will see me as incompetent”. Notably, nine of the 38 respondents who commented in the open-ended text box for this survey question stated that they feel “somewhat independent, but not very confident” as a scientist.

The Thurstonian thresholds for items 1 (classmates and I get the same grades) and 2 (I’m smart enough to be here) all lie within the logit range of the respondent histogram, indicating that respondents endorse every response choice for this item. Similar to items 7 and 10, the relatively large, positive logit value of threshold a for items 1 (-1.01 logits) and 2 (-1.06 logits) indicates that ~25% of respondents strongly disagree with feeling smart enough to be in the chemistry department, while just ~10% of respondents strongly agree with feeling smart enough to be in the chemistry program. This indicates that more sense of belonging than ‘average’ is required for respondents in this population to positively endorse these items. This result echoes sentiments of impostor phenomenon within the respondent population, and aligns well with data from respondent comments, which contain at least 13 mentions of impostor phenomenon. One respondent mentioned that they “often question [their] research and abilities and frequently feel inadequate”, while others stated that they feel not smart enough or “not as good as [their] peers.”

It is interesting to note that items 1, 2, 7, 8, 12, and 13 all target feelings of self-worth and perceptions of others’ views of oneself, and that data from these items suggest that the majority of graduate student and postdoc respondents struggle to relate with viewing themselves as smart, successful, independent, and confident. These data express much of what is known about impostor phenomenon among undergraduates—that feelings of inadequacy in comparison to one’s peers reflect low sense of belonging [38,39], and that sense of belonging is influenced by a feeling that presence and contributions to a community are valued [16,19–21]. However, while undergraduates tend to rely on their class comfort and resulting participation level to feel a sense of belonging [40], graduate students must complete their work independently in order to reach success [37,71]. Pascale et al. suggest that entering graduate school with a purpose to use and apply knowledge in ‘real life’ enhances student investment in courses and overall sense of belonging [46]. In contrast to Pascale’s work, our results suggest that the rigor of graduate coursework, compounded with a lack of faculty mentors and/or negative interactions with one’s faculty advisor(s), negatively impacts sense of belonging among a majority of graduate students.

### Graduate students and postdoctoral researchers sometimes feel happy and accepted

Thurstonian threshold a (-1.83 logits) for item 5 (feel happy and accepted) indicate that all but 2 respondents feel at least *low belonging* with respect to feeling happy and accepted in the Department of Chemistry. Given that no respondents strongly relate with feeling happy and accepted (no respondents score in threshold d; 2.12 logits), data for item 5 indicate that most graduate students and postdocs (~75%) relate with feeling happy and accepted rarely or some of the time (endorsing threshold b; -0.95 logits). In addition, item 4 (have a social support network) threshold a–c values (-1.85, -0.83, and -0.05 logits, respectively) follow the same pattern as for item 5. Threshold d (1.34 logits) lies within the respondent logit distribution. Together, these results indicate that, on average, graduate students feel happy and accepted in relation to their peers sometimes, but not always, and that all respondents feel they have some form of a social support network. This is embodied in comments such as: “friends in your cohort make being here a lot easier”; and “I definitely feel accepted without question. The happiness factor is more a manifestation of…personal academic shortcomings.”

Literature on undergraduate students indicates that their sense of belonging is also tied to giving and receiving comfort and support from peers and friends [40]. While Pascale et. al. found results similar to ours, they also found that graduate students tend to have closer relationships with faculty than they did as undergraduates—which positively contributes to their sense of belonging—because they feel more connected to the faculty as graduate students [46]. While we did find that peer connectedness has a positive impact on sense of belonging among graduate students and postdocs, we found no positive mention of faculty support or friendship in the data for items 4 or 5. It is likely that if graduate students in Berkeley Chemistry had the opportunity to form friendships with faculty, this would have a positive influence on graduate student and postdoc sense of belonging.

Further evidence of the lack of social connectedness between faculty and mentees comes from items 3 (faculty understand the hardships I face) and 11 (not an outsider). Similar to items 1 and 2, the Thurstonian thresholds for these items all lie within the logit range of the respondent histogram, providing evidence that respondents endorse every level of belonging with respect to these items. Approximately 60% of respondents feel negatively or neutral about having faculty they can talk to who understand the hardships they face, and feeling like an outsider (endorse thresholds a and b, but not c or d). Respondent open-ended comments for item 3 mention that not many faculty “share my perspective/experience and [are] who I could go to for advice”, and “faculty are generally selected to be the people who have experienced a lot of success in the academic system…it’s intimidating to talk about feelings of failure”. These comments do not reflect everyone’s sentiments, however—approximately 20% of the respondent histogram lies above threshold d for items 3 and 11, indicating that some respondents do strongly relate with having faculty they identify with, and not feeling like an outsider.

### Graduate students and postdoctoral researchers who identify as members of a URG are less likely to feel a sense of belonging

In open-ended comments for item 3 (faculty understand the hardships I face), respondents also mentioned that it “often feels like not many faculty understand the unique hardships associated with being [part of a URG]”. Respondents wrote more about the lack of URG-identifying faculty members in open-ended responses to item 11 (not an outsider), stating that “my URM identity is represented at a low rate…sometimes I feel like I should express that [diverse] part of me less than I would elsewhere because it isn’t part of the ‘language’ that other people are used to” and that “the department can be quite lonely, especially for people of color”. These findings not only compliment national data suggesting that there is a lack of female and URM-identifying faculty in academic communities [1–9], but they also agree with data from undergraduate populations, suggesting that a lack of mentors and role models negatively impacts sense of belonging among women and URMs for reasons of support and identity [41].

To deepen our understanding of whether graduate students and postdoctoral researchers that identify as members of a URG feel more, less, or the same sense of belonging as majority respondents, we conducted latent regression analysis. Latent regression examines the difference between the mean ability (sense of belonging) of two respondent subpopulations based on independent demographic variables [58,69]. In this case, the populations are URG-identifying and majority respondents. The value of the regression variable based on URG-identity is 0.26 (0.09) logits (p ≤ 0.05). This suggests that respondents from majority groups are significantly more likely to experience a higher sense of belonging than respondents from URGs by an average of 0.26 logits more along the sense of belonging construct (approximately one-sixteenth of the vertical logit scale in Fig 2).

A regression variable was also obtained to estimate the difference in sense of belonging between male- and female-identifying respondent groups. The regression variable is 0.20 (0.10) logits (p ≤ 0.05), suggesting that there is a statistically significant difference between the mean ability (sense of belonging) of men and women. Specifically, that male-identifying respondents are significantly more likely to experience a higher sense of belonging than female-identifying respondents. This result is unsurprising, as national data on undergraduate populations suggests that women and minority populations often experience low sense of belonging in predominantly white and male academic institutions.

### Graduate student and postdoctoral researcher respondents who publish less than their peers are less likely to feel a sense of belonging

**Fig 3:**
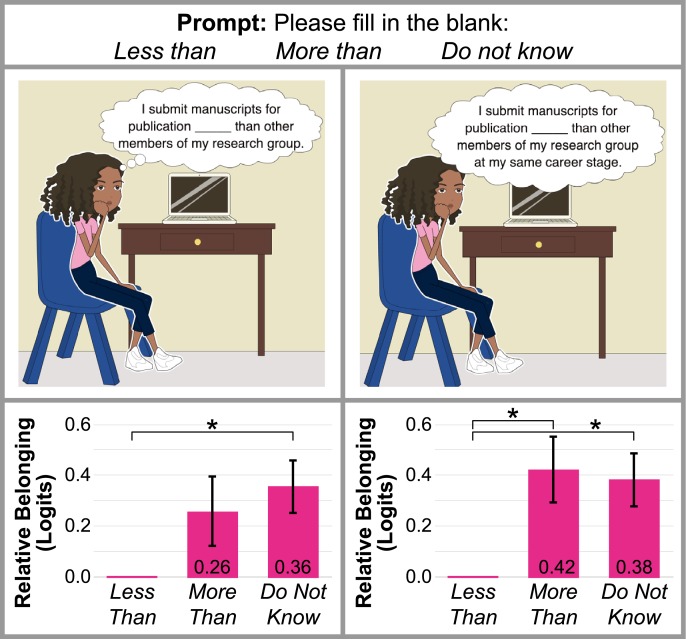
Latent regression variables indicate relationship between sense of belonging and submitting manuscripts for publication. The top row of this table contains the two items from the graduate student and postdoctoral researcher SB survey that assess productivity among peers within a research group, as well as at their same career stage. Graphical comparison of these regression variables is shown in the bottom row, referenced to the logit scores of the population of respondents who feel they publish *less than* their peers. * indicates p ≤ 0.05 for the regression. Regression variables suggest that, in general, respondents who publish less than their peers are less likely to feel a sense of belonging.

Two items in the graduate student and postdoctoral researcher SB survey aim to assess the frequency of publication relative to respondents’ lab mates and same-career stage peers. The goal for these items was to correlate respondent sense of belonging with scholarly productivity *via* latent regression analysis. Many studies gauge graduate student productivity *via* their raw manuscript submission count [47]. Yet, it is important to recognize that that publication rate depends heavily on a student’s field of study, their project, and career stage. For example, physical chemists who spend their graduate careers building and/or troubleshooting an instrument generally publish less than the average synthetic chemist, as noted by one respondent: “not everyone starts in the same place when they start research…when someone inherits a project from a good mentor and the equipment is working, results in this scenario come along much faster than someone who is on a brand new project and builds a lab.” These illustrations and the associated prompt are presented in Fig 3. Additionally, the results of latent regression analysis comparing the sense of belonging logit scores of graduate student and postdoc respondents who feel they publish *less than* or *more than* their peers, or *do not know* whether they publish more or less than their peers are shown.

It is important to note that the regression variables presented graphically at the bottom of Fig 3 can be directly interpreted along the logit scale in Fig 2. Graduate student and postdoctoral researcher respondents who *do not know* if they publish more than or less than other members of their research group have significantly more sense of belonging than respondents who feel they publish *less than* other members of their research group, by 0.36 logits (9% of the logit scale and belonging construct in Fig 2). Respondents who publish *more than* other members of their research group do not have significantly more sense of belonging than respondents who feel they publish *less than* other members of their research group. Populations of respondents who feel they publish *more than*, or *do not know* if they publish more than or less than, other members of their research group at their same career stage have significantly more sense of belonging (by 0.42 and 0.38 logits, respectively) than respondents who feel they publish *less than* their lab mates at their same career stage. These data suggest that students who inherently compare their own research productivity to that of their lab mates—and in particular, notice that they publish less than their same-career-stage-peers—experience more feelings that contribute to low sense of belonging, such as inadequacy, and a lack of independence, confidence, and success.

It is important to note that ‘*at the same rate as’* was not an available response choice for these SB survey items. This was done to avoid an indecisive or “safety net” answer choice. However, it is possible that individuals who do know they publish at the same rate as their lab mates selected *do not know*, which may alter the data in Fig 3. These results provide the first, direct comparison between manuscript submission and graduate student sense of belonging, and thus also solicit further investigation.

### What about faculty? Compiling data to understand sense of belonging among the entire chemistry community

Fourteen Department of Chemistry faculty members completed the SB survey. ConQuest requires at least one response for each possible answer choice in order to fit the data to an item response model, which did not occur with this small sample of faculty respondents. To overcome this issue, the data from the six illustrations that convey the same context in both the faculty and graduate student/postdoc SB surveys were compiled together. The remaining faculty-only item responses were re-scored, such that the 5-point Likert-scale was collapsed to produce a three-level sense of belonging construct: high, neutral, or low belonging. A summary of this new scoring scheme, as well as a summary of the compiled items from both SB surveys, are presented in Table 2.

**Table 2:** Re-scored, faculty-only sense of belonging (SB) construct and scoring guide.

Original SB Score	Original SB Construct	Faculty-Only SB Score	Faculty-Only SB Construct
4	Highest Belonging	2	High Belonging
3	High Belonging		
2	Neutral	1	Neutral
1	Low Belonging	0	Low Belonging
0	No Belonging		

**Fig 4:**
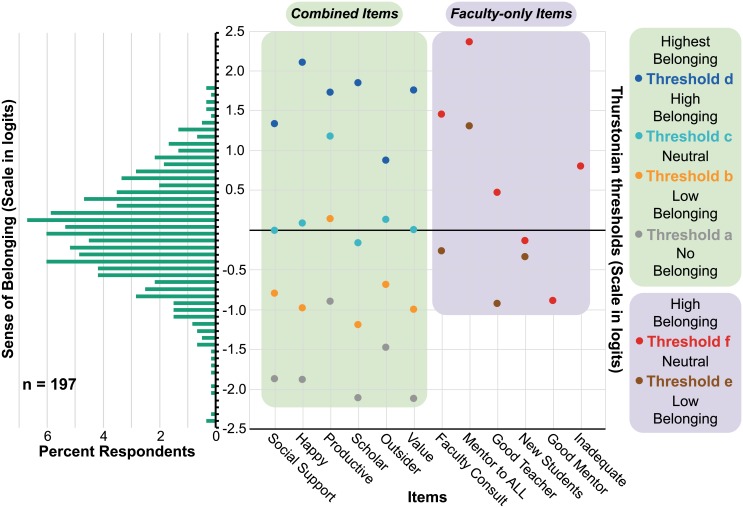
Compiled department of chemistry graduate community Wright map. The histogram on the left-hand side represents the distribution of respondent scores (in logit units) along the sense of belonging construct (y-axis) for the compiled Department of Chemistry graduate student, postdoc, and faculty data. The Thurstonian thresholds for each item are color-coded and shown on the right-hand side; items are labeled according to their short descriptor along the x-axis (key in Table 3). Thurstonian thresholds for faculty-only items are shaded in purple, and those for the complied data are shaded in green.

The resulting Wright map is presented in Fig 4, which can be interpreted similarly to Fig 2. Thurstonian thresholds on the right-hand side of the y-axis are grouped into columns by the item (survey question) they correspond to, and color-coded by threshold letter. Presenting thresholds this way can make it clearer to notice the value of each threshold relative to the others for any single item. The items are labeled along the x-axis according to their short descriptor (key in Table 3). Thurstonian thresholds for items containing combined, graduate community data are shaded in green. Thresholds for faculty-only items are shaded in purple—two thresholds exist for these data, because the 5-point Likert-scale was collapsed into three response choices. For these items, threshold ‘e’ (brown) represents the probability of endorsing a score of 1 (*neutral*) or more on any given item and threshold ‘f’ (red) represents the probability of endorsing a score of 2 (*high belonging*). These thresholds reflect the sense of belonging coding guide in Table 2.

**Table 3:** Descriptor, type, and positively-worded narrative for each item in the graduate student/postdoctoral (GP) and faculty (F) SB surveys.

Item^a^		Descriptor	Item Type	Positively-worded Narrative
GP 4	F 2	Social Support	High SB	I am grateful to have a supportive social network
GP 5	F 3	Happy	High SB	I feel so happy and accepted here
GP 7	F 10	Productive	Low SB	Other [students/faculty] are ***not*** more productive and scientifically successful than I am
GP 8	F 9	Scholar	High SB	I feel like my [audience/peers] see[s] me as a serious scholar!
GP 11	F 7	Outsider	Low SB	I ***do not*** feel like an outsider.
GP 12	F 4	Value	High SB	That was a productive meeting…I’m glad I am valued [by party I am accountable to]
	F 1	Faculty Consult	High SB	I still have that question about my research. I am going to ask the other faculty when they arrive.
	F 5	Mentor to ALL	Low SB	I ***know*** how to be a good mentor to ALL my students.
	F 6	Good Teacher	High SB	I know my students think I’m a good teacher!
	F 8	New Students	Low SB	My colleagues are ***not*** getting more new graduate students than I am.
	F 11	Good Mentor	High SB	I am a good mentor!
	F 12	Inadequate	Low SB	My colleagues are so impressive. It ***does not*** make me feel inadequate.

^a^Rows that are shaded in green provide a summary of the combined SB survey items that produce Compiled Department of Chemistry Graduate Community SB Data. The non-shaded rows represent the re-scored, faculty-only SB survey items.

### Impostor phenomenon affects the entire chemistry community

Within the compiled graduate student, postdoc, and faculty data, ‘as productive and successful as peers’ and ‘not an outsider’ maintain the least negative threshold a logit values (-0.90 and -1.48 logits, respectively). This indicates that just less than one-third of the entire academic community strongly relates with feeling as productive and scientifically successful as their peers—the rest of the respondent population feels less adequate than their peers. In contrast, all of the Thurstonian thresholds for ‘not an outsider’ lie within the logit range of the respondent histogram, and its value for threshold d is smaller than that of any other item (0.87 logits). This indicates that a larger percentage of respondents (~20%) strongly relates with this item than any other item—implying that it is easier for a greater percentage of the population to feel included than relate any other aspect of sense of belonging measured by this survey. In other words, once one stops feeling like an outsider in their community some of the time, it becomes easier to relate with not being an outsider more, or all, of the time.

It is also interesting to note that the Thurstonian thresholds a–c for ‘have a social support network’, ‘viewed as a serious scholar’, and ‘valued’ all have values of 0.00 logits or smaller. Threshold d for these items, however, requires more than ~1–1.5 additional units of belonging to endorse. A similar pattern of Thurstonian threshold values exists for ‘happy and accepted’ (thresholds a–c: ≤ 0.08; d: 2.10 logits). Thus, at least ~50% of graduate students, postdoctoral researchers, and faculty relate with feeling *high belonging* with respect to being valued and viewed as a serious scholar, having a supportive social support network, and feeling happy and accepted in the department. However, less than ~11% of respondents strongly or always relate with those same feelings.

Given that these data reflect what is known about impostor phenomenon among undergraduates, it is clear that impostor phenomenon even affects faculty respondents at Berkeley Chemistry. It is possible that the nature of studying and working at prestigious R1 university makes it difficult to stop comparing oneself to peers that are all equally successful. Regardless of why, it is clear that the entire academic community feels this way. Thus, it is important to brainstorm practical ways to improve these aspects of academic culture to ensure that department members feel happy, valued, welcome, supported, and included. For example, one faculty respondent suggested that “balancing work and family leaves little time for building a social support network at work.” Finding a way to better support department members with a family could be particularly beneficial in achieving this goal. Additionally, increasing discourse within the department about coping with and overcoming academic failures could be a practical way of beginning to normalize feelings of being an impostor.

### Knowing how to mentor all students is the most difficult aspect of sense of belonging for faculty

Thurstonian thresholds e (1.30 logits) and f (2.35 logits) for ‘good mentor to ALL’ have the largest threshold logit values of any faculty-only item—a result which suggests that faculty members find it very difficult to relate with knowing they are a good mentor to students of ALL years, demographics, ages, etc. In fact, not a single respondent scored *high belonging* with respect to this scenario. Respondent comments for this item stood out: “students are a diverse and changing group…some students are harder to reach than others…[being] a good mentor takes a lot of work and reflection…if I am not thinking about how to mentor them all the time, I can’t do my job well”; “not all my students will want to be professors, so how to advise that group is something I’m not super-qualified for. As a male faculty member, I often worry that I do not have the best perspective/experience from which to offer the career advice my female students need.”

The contrast between the Thurstonian threshold logit values for ‘good mentor to ALL’ and ‘good mentor’ is notable. In fact, threshold e for ‘good mentor’ is not even present in Fig 4, and **f** for this item is -0.89 logits. While ~70% of the respondent population lies above the logit value of threshold f for ‘good mentor’, it is not possible to differentiate whether this percentage of the population includes the faculty respondents by eye in Fig 4. However, the raw data for this item (S1 Dataset) confirm that all faculty respondents do relate with being a good mentor. This distinction between being a good mentor to the average graduate student or postdoc versus being a good mentor to ALL students is critical, and likely impacts sense of belonging among mentees who feel they need more substantive mentoring from faculty. In the past, many faculty in our community have noted that they wish they could receive training on how to best communicate with and support students from all backgrounds. It seems that providing training for faculty regarding best practices for engaging in culturally responsive mentoring would be beneficial, and aligns with recommendations from the recent National Academies report on *the Science of Effective Mentoring in STEMM* [72]. Additionally, supplementing students with more (and broader) career advice and professional development opportunities may be useful for all members of our academic community.

### It is difficult for faculty to ask their peers a research question

Data from ‘ask the other faculty about my science’ (threshold e: -0.27 logits; f: 1.45 logits) illustrate that generally, the majority of faculty feel it is difficult to speak with or seek advice from other faculty about their research (few faculty scored *high belonging* with respect to this item). This parallels the finding that grad students and postdocs find it difficult to talk to peers outside of their research group about their science. Open-ended faculty comments for this item, however, demonstrate opposing opinions on this matter. One respondent wrote that “it is useful to get broad advice from other faculty, but most people are going to feel that they should do it on their own”, while a couple of other respondents feel “no difficulty reaching out to colleagues with questions about research”.

### Camaraderie among faculty members

For ‘as many new students as peers’, threshold e has a value of -0.34 logits, and threshold f has a value of -0.14. The close proximity of these values to each other indicate that faculty respondents feel similarly about their ability to recruit new graduate students—specifically, it only takes 0.2 logits of belonging more for faculty to agree that they recruit the same numbers of graduate students as their colleagues, than to disagree or feel neutral about this scenario. In addition, the overall low value for both of these item thresholds indicates that the majority of faculty respondents positively endorsed this item, suggesting that faculty members infrequently feel that they are recruiting fewer new graduate students than their colleagues are. In fact, one respondent commented that “one of the great things about this department [is that] faculty don’t stab each other in the back to get the best grad students…I hear from my colleagues at a number of other universities that this is not the case in their departments, and it is really toxic.”

Support for such camaraderie among faculty is also found in the results for ‘not feeling inadequate’. Note that this item also does not have a value for threshold e in Fig 4 as, in general, all faculty respondents disagree with feeling inadequate compared to their colleagues. The value for threshold f for this item is 0.80 logits, indicating that faculty are either indifferent to or positively endorse not feeling inadequate relative to their colleagues. Respondent open-ended comments for this item provide insight into this result: “some [of my colleagues] are going to win a Nobel Prize…but this does not stop most of us from trying to do great science and take joy in student’s successes”; “it makes me feel lucky to be here for many reasons”. While these data and comments seem contrary to feelings related to impostor phenomenon, it is possible that some faculty members combat such negative feelings with pride for the accomplishments of their colleagues and academic community as a whole.

### Faculty are most likely to feel a sense of belonging; Senior graduate students are least likely to feel a sense of belonging

**Fig 5:**
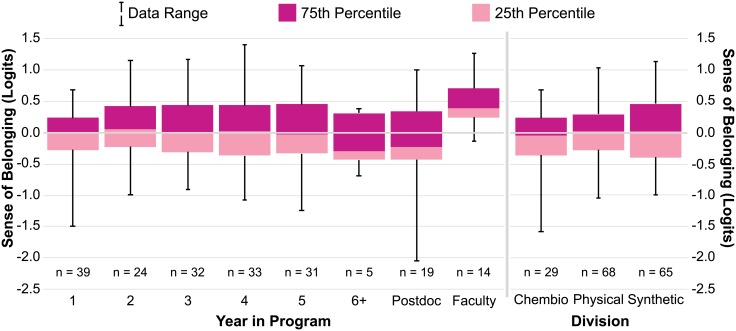
Sense of belonging with respect to academic level and division. Box plots illustrate graduate student, postdoctoral researcher, and faculty sense of belonging (in logits) as a function of academic level and division within the Department of Chemistry. Dark pink represents the third quartile of the overall respondent ability distribution, light pink represents the first quartile, and the intersection of these colored boxes represents the median of the dataset. The number of respondents in each program division includes graduate students and postdoctoral researchers (two students did not select their division).

Sense of belonging among all members of the Department of Chemistry was examined with respect to academic level and division using box plots (presented in Fig 5). Data for sense of belonging (in logits) as a function of year and academic level is presented on the left-hand side of Fig 5, and as a function of division in the program on the right-hand side. The latter contains data from only graduate students and postdoctoral researchers, because the small number of faculty respondents would lead to compromised confidentiality.

Results of this analysis suggest that, in general, there is not much difference in sense of belonging among graduate student respondents in years 1–5 of the chemistry doctoral program. Interestingly, sense of belonging decreases dramatically among senior graduate students—most notably in respondents that are in or past year 6 of their Ph.D. Given that most students in this program leave with a Ph.D. after ~5–5.5 years, it is likely that students who take longer to graduate are left with a decreasingly small social/support network, and thus have a lower probability of feeling like they belong. Postdoctoral researchers also report a similarly low sense of belonging. While it is possible for postdocs to feel high sense of belonging within their field, their high turn-over rate, along with the general lack of a ‘cohort’ when they join the department and a research group, may lead to feelings of isolation and a lack of social connectedness, which negatively impact sense of belonging. In fact, one postdoctoral respondent mentioned that “postdocs do not seem integrated into the department.”

Notably, faculty members report higher overall sense of belonging. This difference in sense of belonging is statistically significant, as indicated by the result of a latent regression carried out to examine the difference between the mean sense of belonging of faculty and graduate student/postdoctoral researcher respondents. The regression variable is 0.88 (0.17) logits (p ≤ 0.05), implying that, on average, faculty have significantly more belonging than graduate students and postdoctoral researcher respondents, by an average of 0.88 logits (approximately one-sixth of the vertical logit scale in Fig 4). The high sense of belonging that faculty respondents feel may be due to the fact that they spend their entire career as a member of the community in which graduate students and postdocs only spend a limited time. It may also be a result of them generally placing more effort and emphasis on finding social support and connectedness among their smaller community of peers, or because faculty members are older and have persisted in academia longer than other respondents.

Analysis of the right-hand side of Fig 5 suggests that sense of belonging among chemical biology (chembio) respondents is slightly below average compared to physical and synthetic chemistry respondents. While this difference is negligible, it is interesting to note that the chemical biology division within Berkeley Chemistry maintains the highest percentage of female- and URM-identifying individuals, compared to the other divisions. Chembio is also the only division that requires its students to participate in research-lab rotations during their entire first year of the program, meaning that these students dive into research, take their candidacy exams, and tend to graduate at least a semester after their peers in other divisions. These differences in program demographics and structure—as well as the structural differences in each year of the Ph.D. program (coursework and candidacy exam timing in years 1 and 2, for example)—provide a foundation for future work investigating the differences in structure and their influence (if any) on graduate student sense of belonging.

## Discussion

Institutions are currently working toward building and maintaining a larger, more diverse community of scientists. However, many of these efforts rely on aggregate, national data regarding underrepresentation of women and URMs to guide their course of action [48,73,74]. In addition, the majority of administrative efforts to foster academic belonging focus exclusively on undergraduate populations, even though graduate students constitute a large percentage of university campuses [46]. Given that the graduate education experience can heavily influence an individual’s decision to pursue a career as a leader in academia, industry, or elsewhere, it is critical to make targeted efforts to identify and address the challenges associated with the academic culture(s) of individual departments that uniquely (and negatively) affect graduate students. Such efforts can also aide in making academic units more welcoming and inclusive to all members [50,73].

The use of a visual narrative and item response theory to quantify sense of belonging among members of the Department of Chemistry at UC Berkeley has enabled a deeper understanding of the factors associated with its academic climate and culture that impact sense of belonging. This study has uncovered specific factors that can be addressed within the graduate academic community in order to improve sense of belonging among all members. Specifically, quantitative analysis of sense of belonging within the entire Department of Chemistry graduate community revealed that members find it most difficult to relate with feeling completely happy, accepted, and valued. In general, respondents also feel that they are not viewed as a serious scholar, and indicate that they do not have a strong social support network. Furthermore, results suggest that graduate students, postdoctoral researchers, and faculty find it difficult to feel independent, confident in their capabilities as a scientist, and as successful and productive as their peers. Community members also struggle with maintaining positive self-worth and perceptions of others’ views of oneself—a result that indicates the existence of impostor phenomenon among members of the academic community at all levels. While these results mirror many of the sentiments expressed by undergraduates experiencing impostor phenomenon [38,39], they also identify factors that had not previously been described in the literature: the impact of being accountable to an advisor and being viewed as a serious scholar on graduate student and postdoc sense of belonging. These factors are largely unique to the graduate community.

In addition, results suggest that graduate student respondents find it commonplace to communicate with their peers about issues they face while teaching (postdoctoral researchers do not teach, and the majority of them did not comment on or respond to this survey item). Given that teaching and pedagogy are early requirements of the Berkeley Chemistry Ph.D. program, it is likely that having a readily-accessible teaching network enables students to feel it is easier, on average, to speak about teaching concerns with their peers and even make efforts to form a community that is concerned with teaching. However, it is also likely that teaching is a low-stakes topic because it is generally not valued as highly as research. The positive contribution of forming a teaching community on graduate student sense of belonging has not been previously discussed in the literature. Yet, it is a factor that likely distinguishes graduate students from undergraduates and may be used by departments to develop a greater support network for entering graduate students.

Particularly surprising is that, while it is not difficult for students and postdocs to ask members of their research group a question about their science or research project, it is very difficult for all members of the chemistry community to talk to peers outside of their research group about science. In fact, most graduate student and postdoc respondents who commented on this item mentioned that they would not feel comfortable asking members of another research group a question, unless they have a good friend in that group. This result is novel, as there is no existing evidence that a lack of collaboration or communication between research groups—whether due to physical proximity of lab spaces, fear of feeling like an impostor, or that it is simply not the ‘norm’ to talk about science with people outside of one’s research group—contributes negatively to sense of belonging among graduate students, postdoctoral researchers, and faculty.

Notably, results suggest that faculty find it extremely difficult to relate with being a good mentor to all their students. Our data also indicate that graduate students and postdocs who identify as members of URGs experience lower sense of belonging than their majority group counterparts. We recognize that the very small number of racial/ethnic minority members of our academic community limits our ability to investigate and address the specific needs of URGs within our academic community, and we plan to conduct interviews and/or focus groups to address this limitation of our study in the future. We also recognize that the faculty data are likely only representative of those respondents who have a vested interest in improving both their approaches to mentoring as well as the overall graduate program. Given that it is well-documented that the lack of female role models and mentors affects sense of belonging among undergraduate women and URMs [41], one immediate step the academic community can take to increase support for students that are currently lacking diverse faculty mentors is to provide training for all faculty members, postdoctoral researchers, and graduate students on how to form fruitful mentoring relationships with students from diverse backgrounds. Education to help academics develop culturally responsive mentoring techniques is a recommendation from the National Academies, as such mentoring can validate students’ identities, help them navigate negative, invalidating experiences, help reinforce their self-efficacy, and greatly increase their likelihood of persisting and thriving in STEM [72].

## Conclusion

This novel methodology and resulting data suggest that sense of belonging at the graduate level is affected by factors that are different from those identified in the literature as affecting undergraduate students [40,43–46]. Sense of belonging has not yet been explored thoroughly within graduate academic communities. Thus, these results deepen the current understanding of the factors within graduate academic culture that impact sense of belonging. In addition, this study presents the first direct, quantitative assessment of sense of belonging as a function of gender, URG identity, academic level, and rates of manuscript submission.

In order to devise practical strategies to address the issues impacting low sense of belonging within a community, the researchers hosted a department-wide information and brainstorming session (cDIBS) [49], which was grounded in these sense of belonging data. This discussion yielded some practical strategies that can be readily implemented, such as: hosting division-wide social events to promote more communication across research groups; establishing a seminar series in which speakers address their scientific hurdles and failures to begin to remove the negative stigma associated with failure; mandating post-candidacy exam meetings with non-advisor faculty to increase the frequency of discourse and mentorship between faculty and students; and continuing to foster productive, evidence-based discussions about the issues faced by our community to ultimately promote action toward positive culture change [49]. The latter includes incorporating topics such as sense of belonging, impostor phenomenon, and failure into the existing monthly Department of Chemistry Diversity and Inclusion Focus Groups (DIFGs), which are grounded in scientific literature and provide a structured, neutral space for participants to explore and engage in challenging conversations [49,75]. Such discussions could enable members of the Chemistry Department to engage in active, open, and honest communication that can facilitate the shift of negative social norms within the academic community.

We believe that a large part of the success of this work to assess academic sense of belonging is due to: (1) the collaborative development of this SB survey; (2) the survey’s tailored visual narrative, which was designed to assess our unique academic climate; (3) the partnership and leadership of graduate students and faculty involved in this initiative; and (4) the intentional dissemination of results to our academic community, in order to raise awareness of the issues within our academic climate that impact sense of belonging. Given that every academic unit has a different climate, culture, and set of needs, values, and goals, there will be no one-size-fits-all survey to perfectly reveal the needs of all academic units [49,76,77]. Other academic units seeking to utilize this same approach should be able to build on this SB survey to address their needs after critical assessment of their status quo and collaborative input from representatives at all academic levels. A customized survey developed in this way will enable the gathering of useful data that can facilitate the implementation of tailored policies and practices to create sustainable, systemic, and positive change within their academic climate [49,76].

The visual narrative and IRT methodology presented herein provide a modifiable blueprint for conducting large-scale assessment of sense of belonging within any doctorate-granting STEM department—particularly the aspects of a graduate program may contribute to and have the biggest impact on sense of belonging. Moreover, IRT analysis can be extended to any subset of respondents based on a target variable, such as year or division in the program, to provide more detailed information regarding the aspects of belonging that are most difficult for that subset of respondents. The calibration of items and respondent abilities on the same scale also makes IRT particularly well-suited to carry out analysis of changes in respondent sense of belonging over time due to institutional changes to improve academic culture. We anticipate that these quantitative methods can be generalized by other academic units seeking to improve the experiences of all scientists. More broadly, this study provides a foundation for stakeholders to build on to improve the graduate education experience and address issues that negatively affect wellness and inclusivity within any doctorate-granting STEM department.

## Supporting information

S1 Text Additional study details. Full sense of belonging visual narrative surveys; survey design and psychometric properties; ConQuest commands for partial credit model analysis.
(PDF)

S1 Dataset Graduate student and postdoctoral researcher respondent dataset. Includes all data necessary for replicating the partial credit model analysis for graduate student and postdoctoral respondents.
(XLSX)
