# Supplementary material for: Sense of belonging within the graduate community of a research-focused STEM department: Quantitative assessment using a visual narrative and item response theory
Source: PLoS One. 2020 May 21;15(5):e0233431. doi: 10.1371/journal.pone.0233431 (PMC7241794; doi:10.1371/journal.pone.0233431)
Supplement: S1 Text — Full sense of belonging visual narrative surveys; survey design and psychometric properties; ConQuest commands for partial credit model analysis. (PDF) [file pone.0233431.s001.pdf]

Supporting Information for

Sense of Belonging within the Graduate Community of a Research-Focused STEM Department: Quantitative Assessment Using a Visual Narrative and Item Response Theory

Christiane N. Stachl*,[a], Anne M. Baranger*,[a,b]

[a]Department of Chemistry, University of California, Berkeley, California 94720-1460, and

[b]Graduate Group in Science and Mathematics Education, University of California, Berkeley, California 94720-1460

*****Christiane N. Stachl

E-mail: cstachl5@berkeley.edu

*Anne M. Baranger

E-mail: abaranger@berkeley.edu

This PDF file includes:

S1 Text

2019 Department of Chemistry Sense of Belonging (SB) Surveys

The final SB surveys include a total of 15 illustrations for graduate students and postdoctoral researchers, and 12 for faculty members. All items are presented in **Fig A**. The prompt for each SB survey illustration/item was the same for both the graduate student/postdoctoral SB survey and the faculty SB survey: *"Please indicate to what extent each of following cartoons relates with your current experience in the Department of Chemistry."* The response choices are as follows: *Do Not Relate; Rarely Relate; Sometimes Relate; Often Relate; Always Relate;* and *Prefer not to respond*.

For illustrations that contain more than one cartoon character (surrounded by asterisks in **Fig A**), the prompt is: *"Please indicate to what extent the experience of the <u>left-most cartoon character</u> relates with your current experience within the Department of Chemistry."* Lastly, the prompt for the 'Frequency of manuscript submission' items in the graduate student/postdoctoral SB survey is: "Please fill in the blank", with the following response choices: *Less than; More than;* or *Do not know*.

Graduate coursework / Self-perceptions

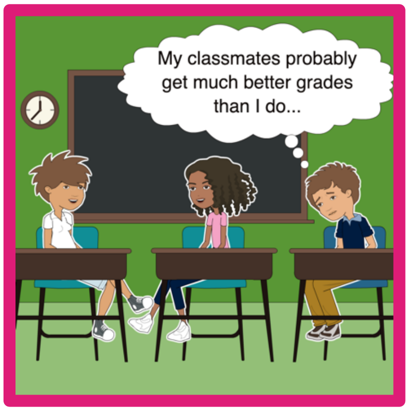

(Low SB)

Graduate coursework / Self-perceptions

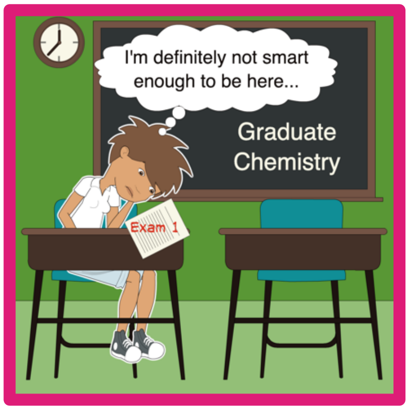

(Low SB)

Interactions with faculty

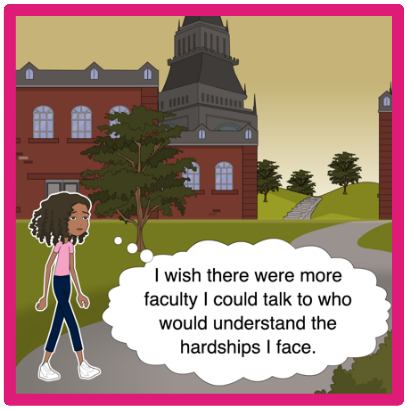

(Low SB)

Social connectedness

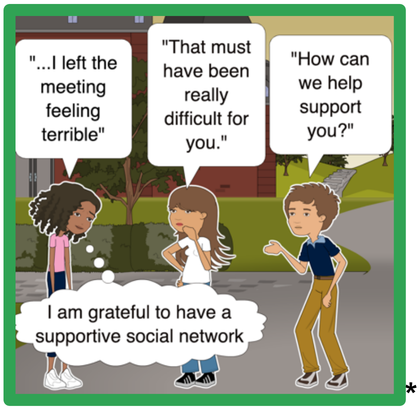

* *

(High SB)

Social connectedness

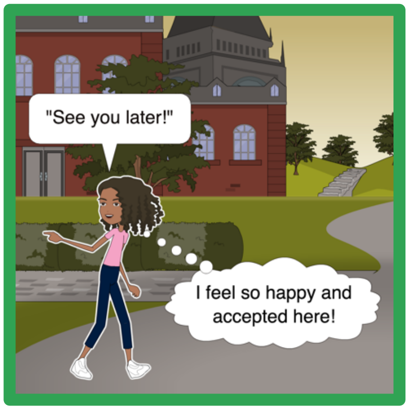

(High SB)

Teaching

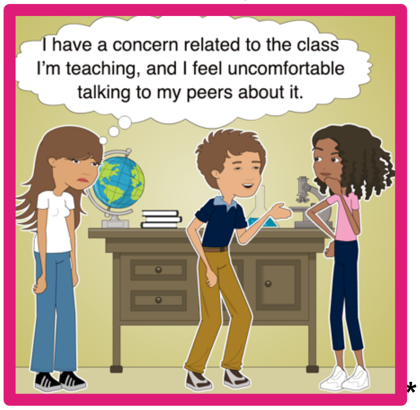

* *

(Low SB)

Self-perceptions (*w.r.t.* undefined measures of success)

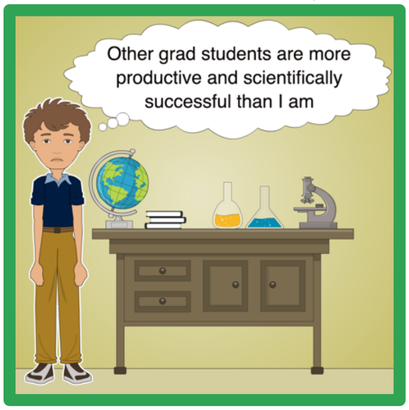

(Low SB)

Self-perceptions (*w.r.t.* undefined measures of success)

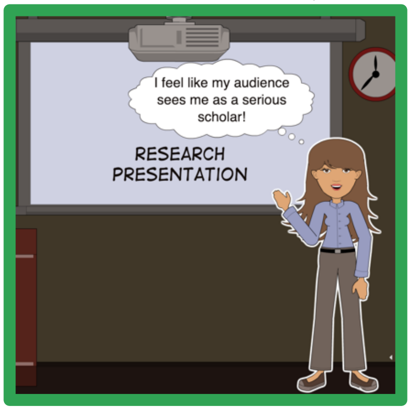

(High SB)

Academic support from peers & mentors

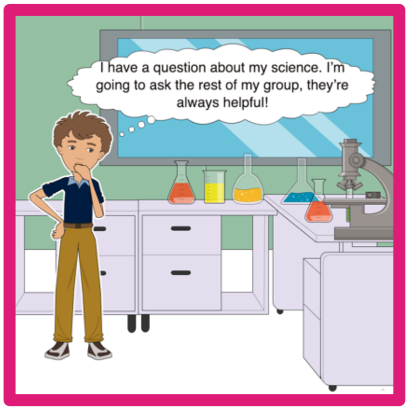

(High SB)

Academic support from peers & mentors

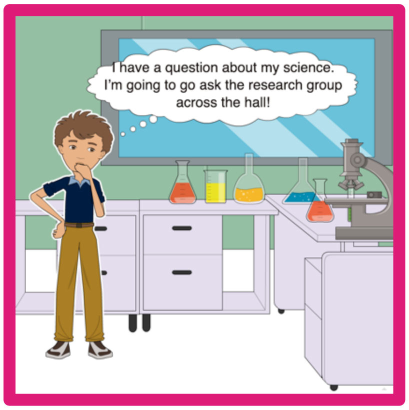

(High SB)

Social connectedness

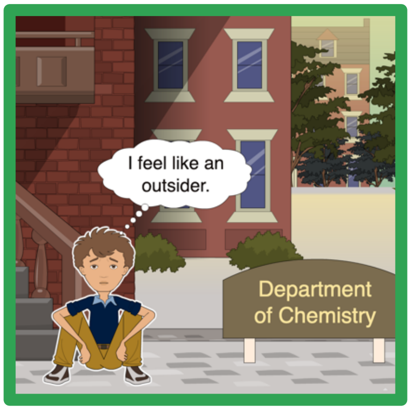

(Low SB)

Interactions with faculty

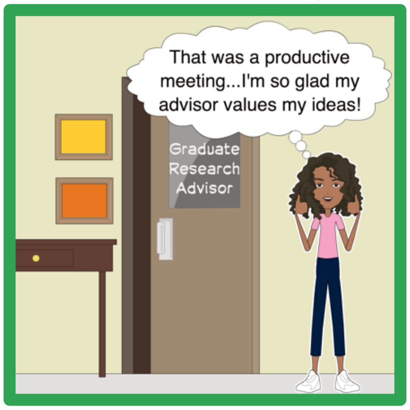

(High SB)

Self-perceptions (*w.r.t.* undefined measures of success)

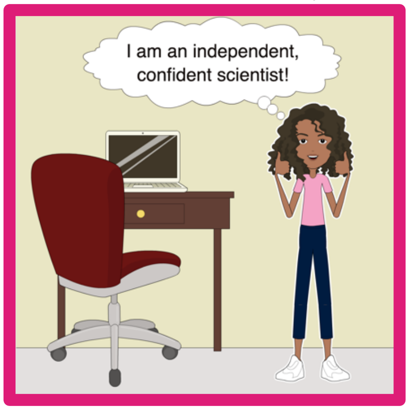

(High SB)

Frequency of manuscript submission

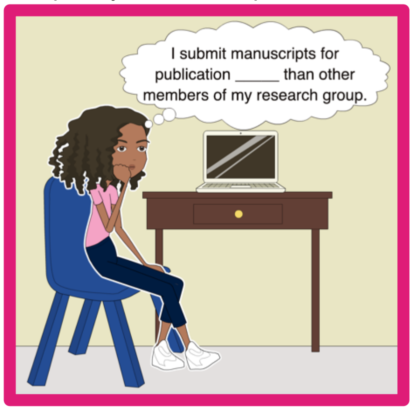

Frequency of manuscript submission

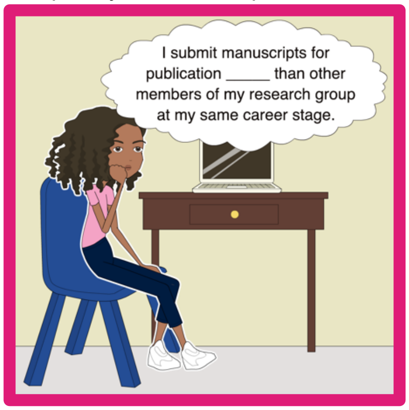

Fig A1. Graduate student and postdoctoral researcher sense of belonging (SB) survey, which is comprised of 15 illustrations. The illustrations that are unique to the graduate student / postdoctoral researcher survey are **outlined in pink**. The six illustrations that are worded to convey the same context in both surveys are **outlined in green**. All illustrations were designed using Pixton Comics Inc.© (pixton.com).

Academic support from peers & mentors

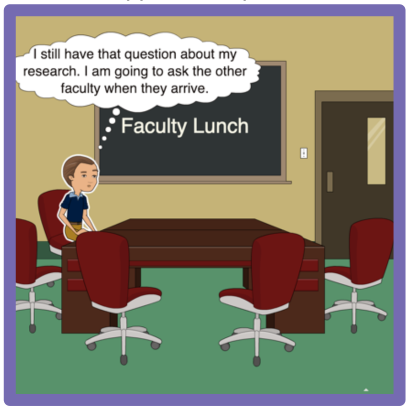

(High SB)

Social connectedness

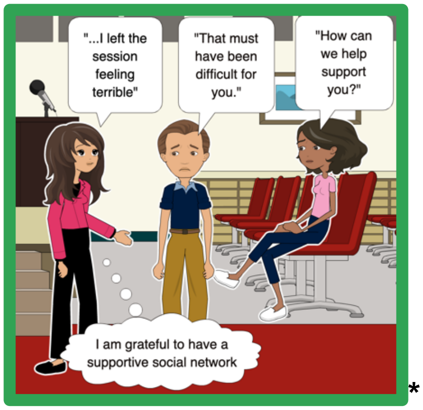

* *

(High SB)

Social connectedness

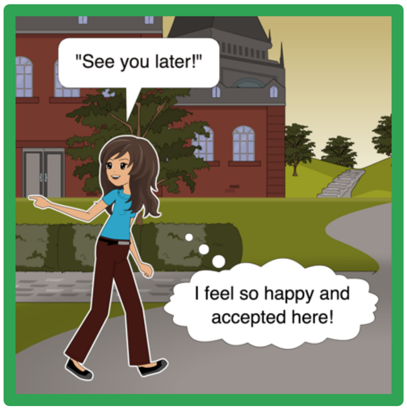

(High SB)

Academic support from peers & mentors

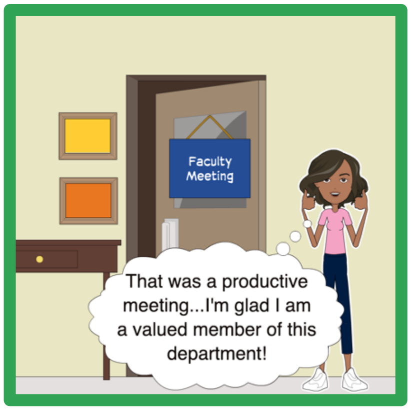

(High SB)

Mentoring

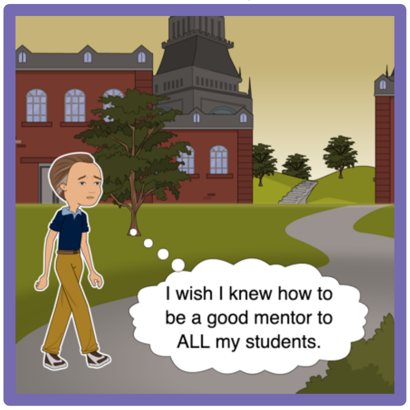

(Low SB)

Teaching

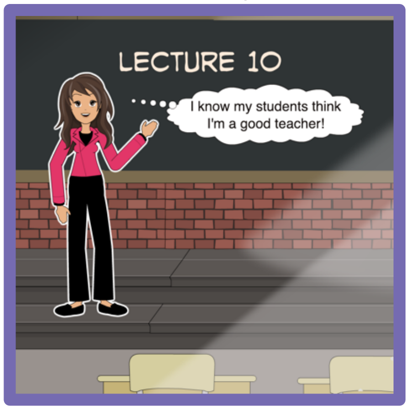

(High SB)

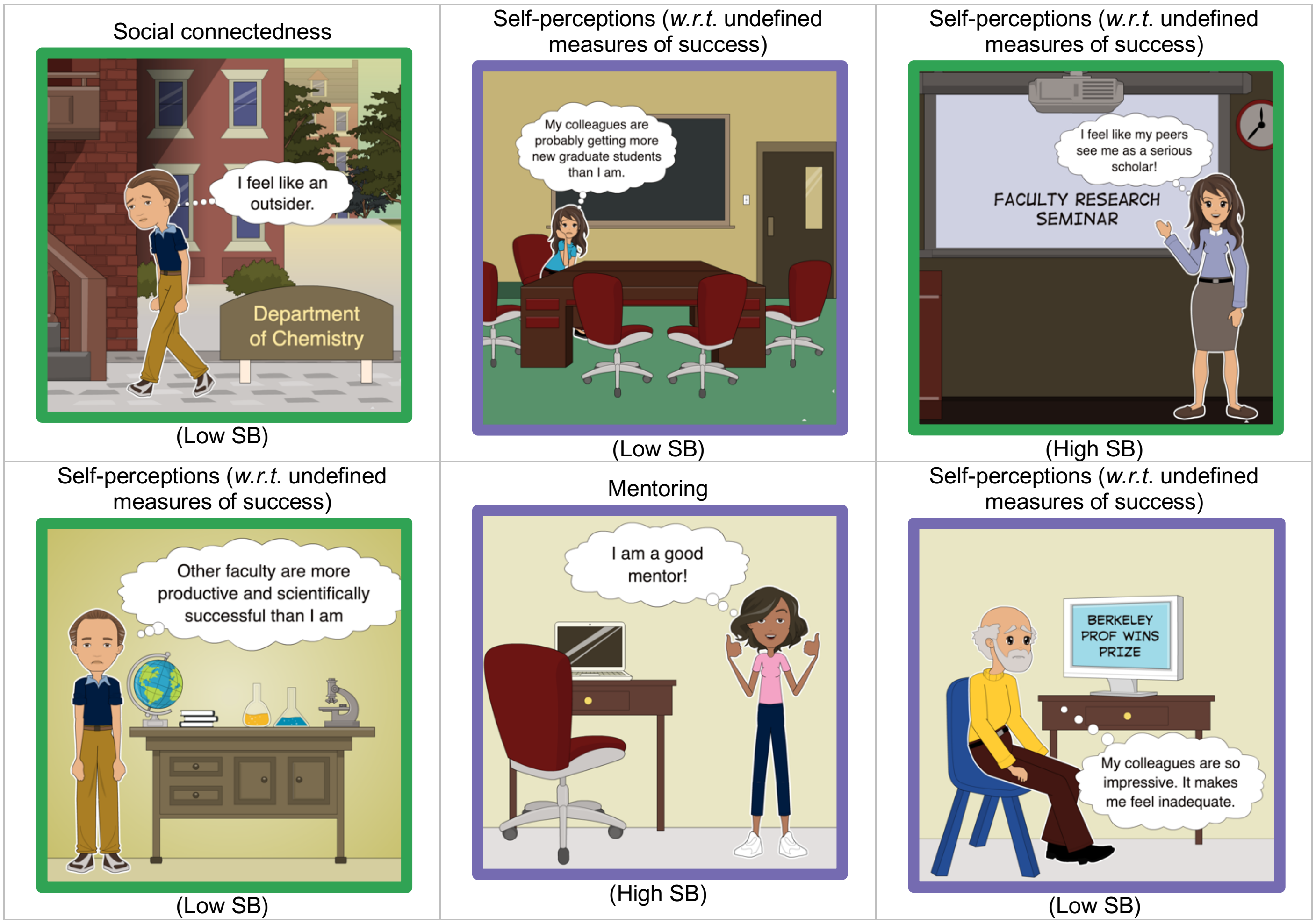

Fig A2. Faculty sense of belonging (SB) survey, which is comprised of 12 illustrations. The illustrations that are unique to the faculty survey are **outlined in purple**. The six illustrations that are worded to convey the same context in both surveys are **outlined in green**. The characters in the faculty survey are portrayed as visually older than those in the graduate student and postdoctoral researcher survey. All illustrations were designed using Pixton Comics Inc.© (pixton.com).

Original and Re-Worded Item Narratives

All previously negatively-worded narratives from low sense of belonging items have been re-worded to aide with interpretation. The original and corresponding re-worded narratives (changes bolded and italicized) are listed in their entirety in **Table A**,

along with the SB survey item descriptors, types, and narratives for the graduate student and postdoctoral (GP) survey, as well as the faculty (F) survey.

Table A. Positively-worded narratives for low SB items.

Item #		Descriptor	Item Type	Narrative	Positively-worded Narrative
**GP 1**		Better Grades	Low SB	My classmates probably get much better grades than I do…	My classmates probably ***do not*** get much better grades than I do…
**GP 2**		Smart Enough	Low SB	I'm definitely not smart enough to be here…	I ***am*** smart enough to be here…
**GP 3**		Hardships	Low SB	I wish there were more faculty I could talk to who would understand the hardships I face	I ***do not*** wish there were more faculty I could talk to who would understand the hardships I face
**GP 4**	**F 2**	Social Support	High SB	I am grateful to have a supportive social network	
**GP 5**	**F 3**	Happy	High SB	I feel so happy and accepted here	
**GP 6**		Teaching	Low SB	I have a concern related to the class I'm teaching, and I feel uncomfortable talking to my peers about it	I ***am comfortable*** talking to my peers about a concern related to the class I'm teaching
**GP 7**	**F 10**	Productive	Low SB	Other grad students are more productive and scientifically successful than I am	Other [students/faculty] are ***not*** more productive and scientifically successful than I am.
**GP 8**	**F 9**	Scholar	High SB	I feel like my audience sees me as a serious scholar!	I feel like my [audience/peers] see[s] me as a serious scholar!
**GP 9**		Group	High SB	I have a question about my science. I'm going to ask the rest of my group, they're always helpful!	
**GP 10**		Hall	High SB	I have a question about my science. I'm going to ask the research group across the hall!	
**GP 11**	**F 7**	Outsider	Low SB	I feel like an outsider.	I ***do not*** feel like an outsider.
**GP 12**	**F 4**	Value	High SB	That was a productive meeting…I'm so glad my advisor values my ideas!	That was a productive meeting…I'm glad I am valued [by party I am accountable to]!
**GP 13**		Independent	High SB	I am an independent, confident scientist!	
	**F 1**	Faculty Consult	High SB	I still have that question about my research. I am going to ask the other faculty when they arrive.	
	**F 5**	Mentor to ALL	Low SB	I wish I knew how to be a good mentor to ALL my students.	I ***know*** how to be a good mentor to ALL my students.
	**F 6**	Good Teacher	High SB	I know my students think I'm a good teacher!	
	**F 8**	New Students	Low SB	My colleagues are probably getting more new graduate students than I am.	My colleagues are ***not*** getting more new graduate students than I am.
	**F 11**	Good Mentor	High SB	I am a good mentor!	
	**F 12**	Inadequate	Low SB	My colleagues are so impressive. It makes me feel inadequate.	My colleagues are so impressive. It ***does not*** make me feel inadequate.

Demographic Questions (Graduate Students and Postdoctoral Researchers):

- What year are you in the Chemistry Ph.D. program at UC Berkeley?
- Did you enter the program as a physical chemistry, synthetic chemistry or chembio student?
- Did you enter the program on an F-1 or J-1 (or other) student visa?
- Optional: With which gender do you most identify? (*Answer choices: Male; Female; Nonbinary*)
- Optional: Do you consider yourself part of an underrepresented group? (*The term Underrepresented Group (URG) is meant to include, but is not limited to individuals: That identify as female; From underrepresented racial, religious, ethnic, sexual orientation, and international groups; With disabilities (defined as those with a physical or mental impairment that substantially limits one or more major life activities); and With low socio-economic status)*

Demographic Question (Faculty):

- I am a _____ faculty member. (*Answer choices: Physical, nuclear, or theoretical chemistry; Synthetic chemistry or chemical biology*)

Additional Results: Respondent Populations

Table B. Sense of belonging (SB) survey respondent populations.

Respondent Population	Number of Respondents	Total Population	Percent Respondents
Graduate Students	164	335	49%

Postdoctoral Researchers	19	85	22%
Faculty Members	14	66*	21%
*There have been 3 new faculty hires in the Department of Chemistry since the release of this survey			

Survey Design and Psychometric Properties: Validity evidence

There are a variety of means by which the validity of an instrument—whether it measures what it is intended to—can be determined. The five aspects of validity evidence are: evidence based on instrument content, response processes, internal structure, relations to other variables (external validity), and consequences (1).

Validity evidence based on instrument content is determined by providing evidence of the relationship between the content of this instrument and construct it was designed to measure. This comes from Wilson's "Four Building Blocks" (1): defining the construct, carrying out items design, and determining both the outcome space and the Wright map. The construct was defined by identifying factors that contribute to sense of belonging from literature and by talking to members of the Chemistry Community at UC Berkeley; items were designed based on those dimensions and scrutinized *via* extensive item paneling, focus groups, and think aloud interviews among members of the Chemistry and Education Communities at UC Berkeley; scoring was determined by the construct map (**Table 1**, main text) after piloting the survey; and a Wright map is used to relate the data to the construct, and define a scale by which to measure sense of belonging.

Validity evidence based on response process was provided as the survey illustrations were designed, based on many rounds of think aloud interviews with and

exit surveys from graduate students and faculty members in the Department of Chemistry. This provided very helpful feedback about the illustrations, particularly regarding character demographics, how easy the items are to understand, as well as how relatable, redundant, and/or difficult the items are. This feedback was incorporated into the final version of the survey illustrations.

Additionally, the pilot version of this survey contained only open-ended items rather than Likert-scale items because (1) we were not certain of the extent to which respondents would relate with each illustration, and (2) open-ended questions allow for a greater variety of responses and insight into the justification of each respondent's level of relation to each item. Based on the pilot data, the responses contained five distinct levels of agreement. Thus, we use 5 Likert-scale items in this study.

Validity evidence based on internal structure at the instrument level is provided by the Wright maps (**Figs 2** and **4**, main text), which offer evidence that the empirical data obtained supports the theoretical expectations of the construct map.

Based on the Wright map in **Fig 2** (main text), it is evident that the range of instrument items logit values spans the entire distribution of respondent logit values, even though there are also a number of item thresholds that are located above the top respondent logit value. This indicates that the survey 'difficulty' is appropriately suited to measure sense of belonging within this respondent population. The Wright maps in **Figs 2** and **4** (main text) also suggest that the majority of respondents relate with (score at or endorse) lower levels of belonging more than the highest levels of belonging for all items—this is seen through the 'ordering' of thresholds from A-D/E-F for every item. **Fig 4** (main text) illustrates that compiling the data from the entire chemistry department still

resulted in a Wright map in which the range of instrument item logit values spans the entire distribution of respondent logit values, such that both sense of belonging surveys are appropriately suited to measure sense of belonging within our academic community.

The respondent ability histograms in **Figs 2** and **4** are also symmetric, implying that neither data set is skewed or bimodal with respect to the distribution of respondent abilities. Respondent abilities in **Fig 2** range from approximately 1.5 to -2.5 logits, and most respondents falling within two standard deviations from the mean (-0.003 ± 0.554 logits). Respondent abilities in **Fig 4** range from ~1.75 to -2.5 logits. Most respondents fall within three standard deviations from the mean (-0.002 ± 0.560 logits).

Survey Design and Psychometric Properties: Reliability Evidence

In order to investigate the consistency with which the instrument measures respondent ability along the construct, however, the “coefficient of internal consistency”, or “person separation reliability” (1) needs to be calculated. This measure of survey reliability is similar to Cronbach’s α, and provides information about the proportion of variance that the model accounts in estimating respondent ability along the construct (1). The reliability of partial credit model analysis carried out on the graduate student and postdoctoral researcher data is ***0.799***. This value indicates an acceptable consistency of the items to measure respondent ability (2,3). The Wright map and this high consistency coefficient, indicate that the items in this survey relate to each other and do provide a reliable measure of the construct.

The reliability of partial credit model analysis on the combined community data is ***0.794***—very similar to that of just the graduate student and postdoctoral researcher

survey. This value also indicates an acceptable consistency of the items to measure respondent ability, and that the items in both the grad/postdoc and faculty surveys relate to each other and do provide a reliable measure of the construct.

Item Response Analysis: Partial Credit Model vs. Rating-Scale Model

Another adjacent-category IRT model is the rating-scale model. The primary difference between the partial credit and the rating-scale models is the parameterization techniques used to determine item difficulty parameters. The partial credit model enables each item to maintain an independent rating scale structure, while the rating-scale model defines a constant rating scale and only one set of difficulty parameters for each item (3–6). In this study, the partial credit model was used because it allows for variation in item parameters, which enables a more informative and deeper analysis of the items in this survey. Specifically, which levels of the construct *within each item* are the most least difficult for respondents to achieve. Such item parameters are very useful data to have (in addition to the overall item difficulties calculated by the model) to quantify sense of belonging within the chemistry graduate community along the dimensions of sense of belonging presented in the survey.

Additionally, both the partial credit and rating-scale models were used to obtain model parameters based on the data gathered in this study. The Chi Squared Distribution table was used to compute the difference between the partial credit and rating-scale model deviances and degrees of freedom. The X^2 value is significant at the 0.001 level of significance, thus indicating that the “larger” partial credit model—with

more freely estimated parameters—fits the graduate student and postdoctoral respondent data better than the rating-scale model (**Table C**).

Table C. X^2 value comparison between partial credit and rating-scale model analysis of the graduate student and postdoctoral respondent data.

	Partial Credit Model	Rating-Scale Model	Difference ($X^2_{diff} = X^2_s - X^2_l$)
**Final Deviance**	6210.5	6340.0	~130
**Number of Parameters**	53.0	17.0	-36 ($\alpha_{0.001}$ = 67.9)

Standard Error of Measurement

The Logit estimation method used for persons throughout this paper to determine respondent abilities according to their pattern of item responses and the estimated partial credit model item parameters is EAP (*expected a posteriori*) (7). the ‘ability’ associated with each respondent is measured only as an estimate of their true sense of belonging, expressed via their EAP score. The standard error of measurement (EAP variance) associated with each ability provides information about how accurate the EAP estimates are. The smaller the variance, the greater the accuracy and reliability of the estimated ability. **Fig B** (EAP vs. EAP Variance) shows that the standard error of measurement is fairly low (≤0.35). There are a couple of anomalous data points with high EAP error associated with the respondents whose proficiencies are -2.05, -1.49, and 0 logits. Overall, this plot is very flat due to the coverage of thresholds by the respondent population, suggesting that this survey is useful for assessing sense of belonging.

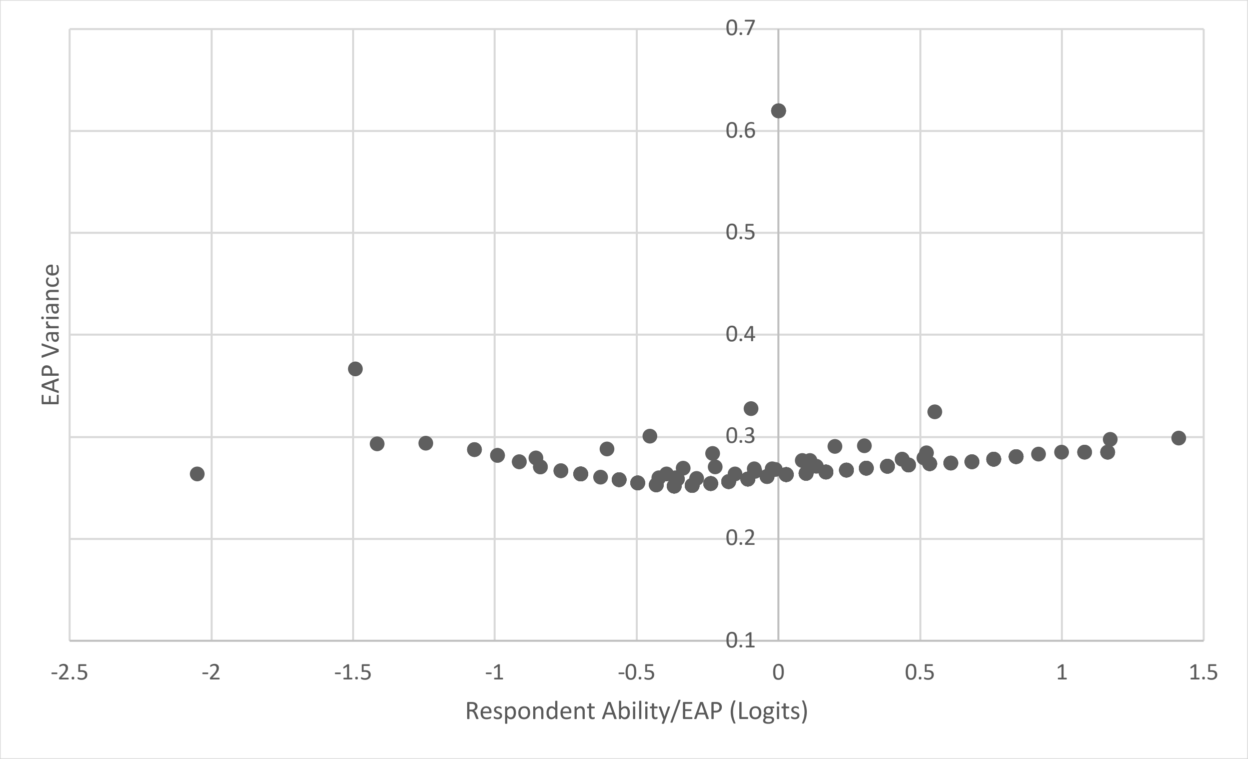

Fig B. Plot of respondent EAP scores vs. EAP error.

ConQuest Commands for Partial Credit Model Analysis

Graduate Student and Postdoctoral Dataset:

Title 2019SBGradPostdoc;

datafile 2019SBGradPostdoc.txt;

format responses 1-13;

codes 0,1,2,3,4;

set constraints = cases;

model item + item*step;

estimate!stderr=quick;

export parameters >> ‘filename’.txt;

```
show cases !estimates=eap >> 'filename'.txt;
show cases !estimates=mle >> 'filename'.txt;
show cases !estimates=wle >> 'filename'.txt;
itanal >> 'filename'.txt;
show !tables=1:2:3:4:5:7, estimate=latent  >> 'filename'.txt;
reset;
```

Code is the same for Compiled Data.

All data necessary for replicating partial credit model analysis for graduate student and postdoctoral respondents is included in additional Supporting Information files and is coded according to scheme in **Table 1** (main text). Note: missing data is coded as "."

ConQuest Command for Partial Credit Model Latent Regression Analysis (Graduate Student and Postdoctoral Dataset):

```
Title Regress Dataset;
datafile 2019_regress.txt;
format responses 1-13 R1 14; *regression variable in column 14*
codes 0,1,2,3,4;
set constraints = cases;
model item + item*step;
regression R1;
estimate!method=quadrature, stderr=quick;
export parameters >> 2019_regress_PCS_parms.txt;
export reg_coefficients >> 2019_regress_PCS_reg.txt;
```

```
show parameters!table=7 >> 2019_regress_PCS_levels.txt;
show !estimates=latent >> 2019_regress_PCS_showfile.txt;
itanal >> 2019_regress_PCS_itn.txt;
show cases ! estimates=mle >> 2019_regress_PCS_mles.txt;
show cases ! estimates=eap >> 2019_regress_PCS_eaps.txt;
show cases ! estimates=wle >> 2019_regress_PCS_wles.txt;
reset;
```

Additional Results of Item Response Analysis: Item Difficulties

The partial credit model outputs item difficulties, in addition to respondent abilities and Thurstonian thresholds. Calculating abilities and item parameters for every respondent and item enables partial credit model analysis to be completed at the respondent, item, or institutional level, or all of the above. The item difficulties produced from partial credit model analysis are summarized in **Tables D and E**. To directly compare item difficulties for the positively- and negatively-worded items in each SB survey, the negatively-worded narratives are re-worded (changes bolded and italicized) to aide with interpretation and reflect the scoring scheme in **Table 1** (main text).

Table D. Item difficulties and MNSQ fit statistics from the graduate student and postdoctoral (GP) researcher survey, calculated by partial credit model analysis.

Item #	Descriptor	Positively-worded Narrative	Difficulty (Logits)	Weighted Fit (MNSQ + CI)
**GP 1**	Better Grades	My classmates probably ***do not*** get much better grades than I do…	0.185 ± 0.070	1.07 (0.82, 1.18)
**GP 2**	Smart Enough	I ***am*** definitely smart enough to be here…	0.188 ± 0.071	0.82 (0.82, 1.18)
**GP 3**	Hardships	I ***do not*** wish there were more faculty I could talk to who would understand the hardships I face	-0.019 ± 0.069	0.97 (0.82, 1.18)
**GP 4**	Social Support	I am grateful to have a supportive social network	-0.345 ± 0.078	1.20 (0.81, 1.19)
**GP 5**	Happy	I feel so happy and accepted here	-0.115 ± 0.086	0.85 (0.80, 1.20)
**GP 6**	Teaching	I have a concern related to the class I'm teaching, and I ***am comfortable*** talking to my peers about it	-1.283 ± 0.097 (**least** difficult item)	1.10 (0.73, 1.27)

**GP 7**	Productive	Other grad students are ***not*** more productive and scientifically successful than I am	0.662 ± 0.082 (**most** difficult item)	1.00 (0.80, 1.20)
**GP 8**	Scholar	I feel like my audience sees me as a serious scholar!	-0.289 ± 0.091	1.00 (0.79, 1.21)
**GP 9**	Group	I have a question about my science. I'm going to ask the rest of my group, they're always helpful!	-1.150 ± 0.088	1.02 (0.77, 1.23)
**GP 10**	Hall	I have a question about my science. I'm going to ask the research group across the hall!	0.490 ± 0.074	1.29 (0.81, 1.19)
**GP 11**	Outsider	I ***do not*** feel like an outsider.	-0.227 ± 0.071	0.82 (0.82, 1.18)
**GP 12**	Value	That was a productive meeting…I'm so glad my advisor values my ideas!	-0.308 ± 0.085	1.06 (0.80, 1.20)
**GP 13**	Independent	I am an independent, confident scientist!	0.067 ± 0.087	0.84 (0.80, 1.20)

For the graduate student and postdoctoral (GP) researcher survey, item difficulties range from approximately -1.3 to 0.7 logits. Item 6 (teaching) has the most negative logit value, indicating that this is the least difficult item for respondents to relate with. In contrast, item 7 (productive) has the most positive logit value, which indicates that it is the most difficult item for respondents to endorse. Items 10 (hall), 2 (smart enough), 1 (better grades), and 13 (independent) are the next most difficult items—indicating that respondents find it difficult to feel confident in their own knowledge, independence and confidence, relative to their peers.

The weighted infit mean square (MNSQ) and corresponding 95% confidence interval (CI)—a measure of each item's 'fit' to the model used for measurement—is also reported for each item in **Table D**. The desired range for MNSQ values is between 0.77 and 1.33 (2,3,7,8). In general, all of the items in this survey have MNSQ values that lie within the desired range, suggesting that each item's data fit the partial credit model appropriately, and that there is an appropriate amount of variation in responses associated with these items.

Item difficulties obtained from partial credit model analysis of the compiled graduate community and faculty-only data are reported in **Table E**. The item difficulties for the compiled data range from approximately -0.9 to 1.8 logits.

Table E. Item difficulties and MNSQ fit statistics from the compiled graduate community data, calculated by partial credit model analysis

Descriptor	Difficulty (Logits)	Weighted Fit (MNSQ + CI)
Social Support	-0.335 ± 0.075	1.23 (0.82, 1.18)
Happy	-0.166 ± 0.083	0.88 (0.81, 1.19)
Productive	0.521 ± 0.076	0.97 (0.81, 1.19)
Scholar	-0.401 ± 0.087	0.95 (0.80, 1.20)
Outsider	-0.293 ± 0.069	0.83 (0.82, 1.18)
Value	-0.339 ± 0.083	1.08 (0.81, 1.19)
Faculty Consult	0.589 ± 0.402	1.14 (0.44, 1.56)
Mentor to ALL	1.828 ± 0.465 (**most** difficult item)	1.03 (0.10, 1.90)
Good Teacher	-0.227 ± 0.395	1.03 (0.39, 1.61)
New Students	-0.238 ± 0. 349	1.42 (0.31, 1.69)
Good Mentor	-0.886 ± 0.665 (**least** difficult item)	0.99 (0.25, 1.75)
Inadequate	0.798 ± 0.559	0.98 (0.73, 1.27)

'Mentor to ALL' has the most positive logit value—this faculty-only item has a much greater logit value than the most difficult item for the grad/postdoc-only survey. In contrast to this result, the item difficulty for 'good mentor' indicates that faculty do not find it as hard to agreeing with being a good mentor, in a broad sense. Moreover, 'teaching' maintains the most negative logit value, while 'productive' has the largest difficulty value. It is also interesting to note that 'faculty consult' and 'hall' both have large, positive item difficulties.

The weighted infit mean square (MNSQ) value and corresponding 95% confidence interval (CI) for each item is also reported in **Table E**. In general, all of the MNSQ values for item location lie in the desired range (0.77 < MNSQ < 1.33). There is one exception to this—'new students' has a MNSQ value of 1.42, which is likely caused

by the small number of responses associated with this item (93% of the faculty members that responded to this item scored highest belonging, which suggests that there was not much variation in the responses to this item). A larger respondent population could remedy this MNSQ exception.

Differential Item Functioning: Graduate Student and Postdoctoral Survey

Whenever item response theory is used to assess the a latent trait among a population of respondents, it is assumed that the population is qualitatively homogenous, or “invariant”, such that the ability estimates and item parameters calculated by the model do not depend on a particular sample of respondents (3,9). To ensure that this assumption applies to a given survey and population, the items need to be tested for differential item functioning (DIF). DIF is a violation of the invariance assumption—its existence within a set of items indicates that respondents with equal ability (i.e., sense of belonging) have different probabilities of endorsing any given item (3,9). DIF is traditionally used to assess whether a survey can differentiate on the basis of demographic differences between respondents (culture, ethnicity, gender, etc.).

DIF was assessed for this sense of belonging survey, to ensure that the items are not biased toward a group of respondent subgroups (male vs. female vs. URM-identity, for example), such that groups of respondents with the same ability/sense of belonging perform differently on any given item (1,10,11). This analysis was carried out only on the graduate student and postdoctoral researcher survey—there are not enough

faculty members that identify as female or URMs to validate collection of faculty demographic information. **Table F** provides a summary of the DIF variables.

Table F.

Demographic Variable	DIF Variable
Gender	0.100 ± 0.032
URG-identity	0.138 ± 0.032
Visa status	0.052 ± 0.032

The DIF variables for gender, URG-identity, and visa-status are not statistically significant, based on the recommended rule for the effect sizes of DIF statistics, which suggests that a logit difference value less than 0.426 is “negligible” DIF (1,10,11). This suggests that, on average, male-, non-URG-, and non-international- identifying respondents perform negligibly higher on this survey. DIF analysis was also performed at the item level, and similar results were obtained. Overall, such results indicate that this survey is not biased toward any particular population.

References

1. Wilson M. Constructing measures: An item response modeling approach. Mahwah, NJ, US: Lawrence Erlbaum Associates Publishers; 2005.
2. Bond TG, Fox CM. Applying the Rasch Model. 2nd ed. New York: Psychology Press; 2007.
3. Wright B, Masters G. Rating scale analysis. Chicago, IL: MESA Press; 1982.
4. Kennedy CA. Constructing Measurement Models for MRCML Estimation: A Primer for Using the BEAR Scoring Engine. Berkeley Eval Assess Res Cent.; 2005.
5. Masters GW, Wright BD. Model for Partial Credit Scoring. Chicago, IL: University

of Chicago, Department of Education, Statistical Laboratory; 1981.

6. Masters GN. A rasch model for partial credit scoring. Psychometrika. 1982 Jun;47(2):149–74.

7. Embretson SE, Reise SP. Item Response Theory for Psychologists. Mahwah, NJ: Lawrence Erlbaum Associates, Inc.; 2000.

8. Wu M, Adams RJ. Properties of Rasch Residual Fit Statistics. J Appl Meas. 2013;14(4):339–55.

9. Objective measurement III: Theory into practice. Engelhard G, Wilson M editors. Norwood, NJ; 1996.

10. Longford NT, Holland PW, Thayer DT. Stability of the MH D-DIF statistics across populations. In: Holland PW, Wainer H, editors. Differential item functioning BT - Differential item functioning. Hillsdale, NJ, US: Lawrence Erlbaum Associates, Inc.; 1993. p. 171–96, Chapter xv, 453 Pages.

11. Paek I. Investigations of differential Item functioning: Comparisons among approaches, and extension to a multidimensional context. University of California, Berkeley; 2002.
